# Supplementary material for: Real-time forecasting of data revisions in epidemic surveillance streams
Source: PLoS Comput Biol. 2025 Nov 20;21(11):e1013709. doi: 10.1371/journal.pcbi.1013709 (PMC12646461; doi:10.1371/journal.pcbi.1013709)
Supplement: S1 Appendix — Shown are DelphiRF count revision forecasts for CHNG outpatient insurance claims data at a reporting lag of 7 days across U.S. states. The model is re-trained every 30 days using training windows of 180 and 365 days, respectively. The figure format follows that of Figs 6 and 7. (PDF) [file pcbi.1013709.s002.pdf]

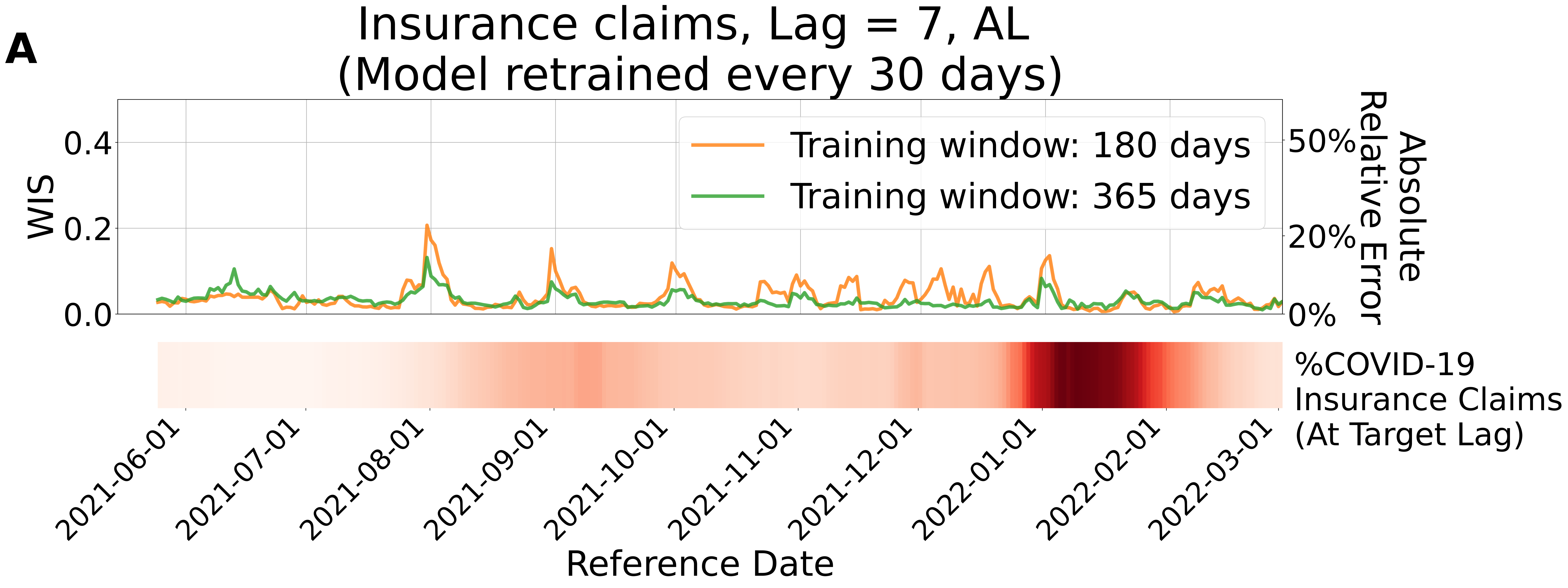
A
Insurance claims, Lag = 7, AL
(Model retrained every 30 days)
WIS
0.0
0.2
0.4
Absolute Relative Error
0%
20%
50%
Training window: 180 days
Training window: 365 days
%COVID-19 Insurance Claims (At Target Lag)
2021-06-01
2021-07-01
2021-08-01
2021-09-01
2021-10-01
2021-11-01
2021-12-01
2022-01-01
2022-02-01
2022-03-01
Reference Date

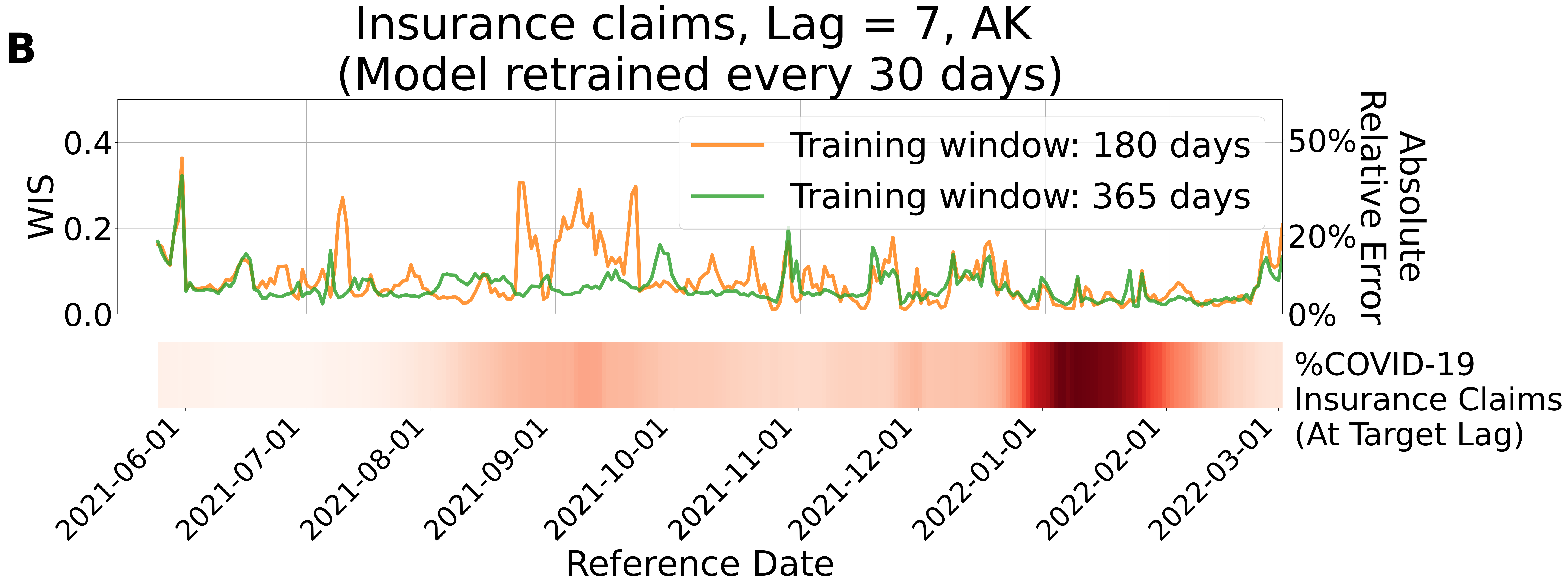
B
Insurance claims, Lag = 7, AK
(Model retrained every 30 days)
WIS
0.0
0.2
0.4
Absolute Relative Error
0%
20%
50%
Training window: 180 days
Training window: 365 days
%COVID-19 Insurance Claims (At Target Lag)
2021-06-01
2021-07-01
2021-08-01
2021-09-01
2021-10-01
2021-11-01
2021-12-01
2022-01-01
2022-02-01
2022-03-01
Reference Date

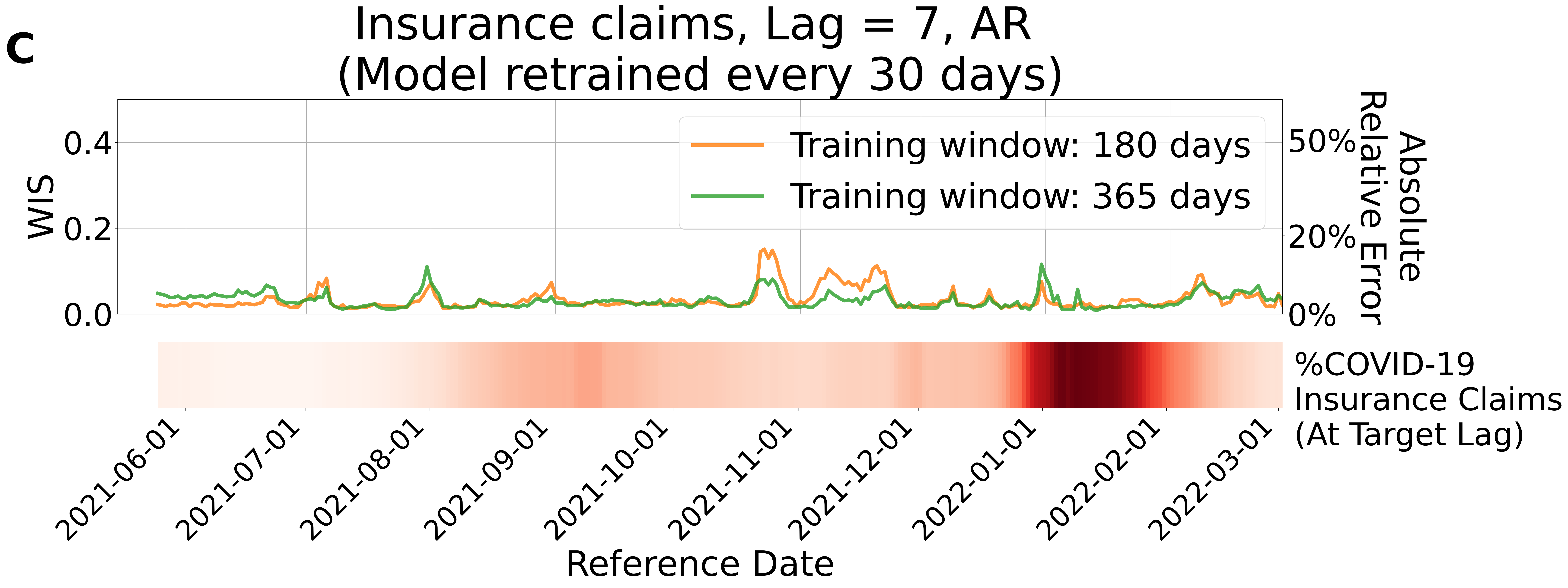
c
Insurance claims, Lag = 7, AR
(Model retrained every 30 days)
WIS
0.4
0.2
0.0
50%
20%
0%
Absolute Relative Error
Training window: 180 days
Training window: 365 days
%COVID-19 Insurance Claims (At Target Lag)
2021-06-01
2021-07-01
2021-08-01
2021-09-01
2021-10-01
2021-11-01
2021-12-01
2022-01-01
2022-02-01
2022-03-01
Reference Date

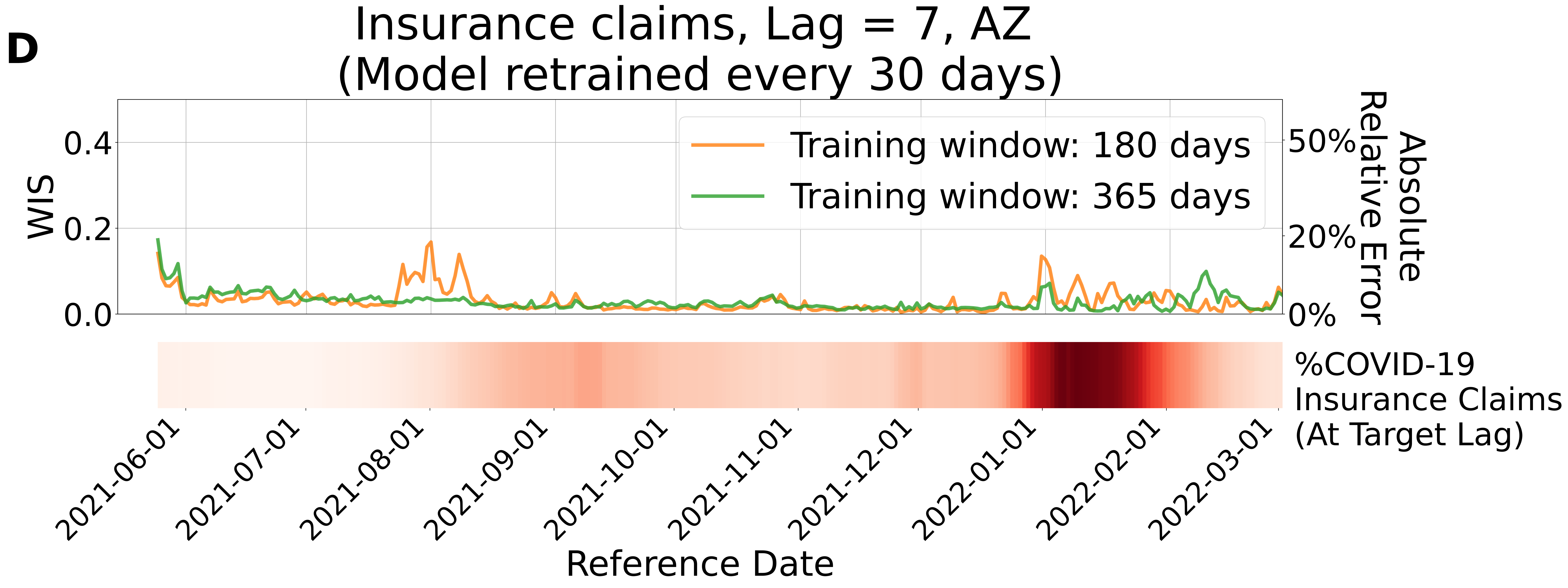
D
Insurance claims, Lag = 7, AZ
(Model retrained every 30 days)
WIS
0.0
0.2
0.4
Absolute Relative Error
0%
20%
50%
Training window: 180 days
Training window: 365 days
%COVID-19
Insurance Claims
(At Target Lag)
2021-06-01
2021-07-01
2021-08-01
2021-09-01
2021-10-01
2021-11-01
2021-12-01
2022-01-01
2022-02-01
2022-03-01
Reference Date

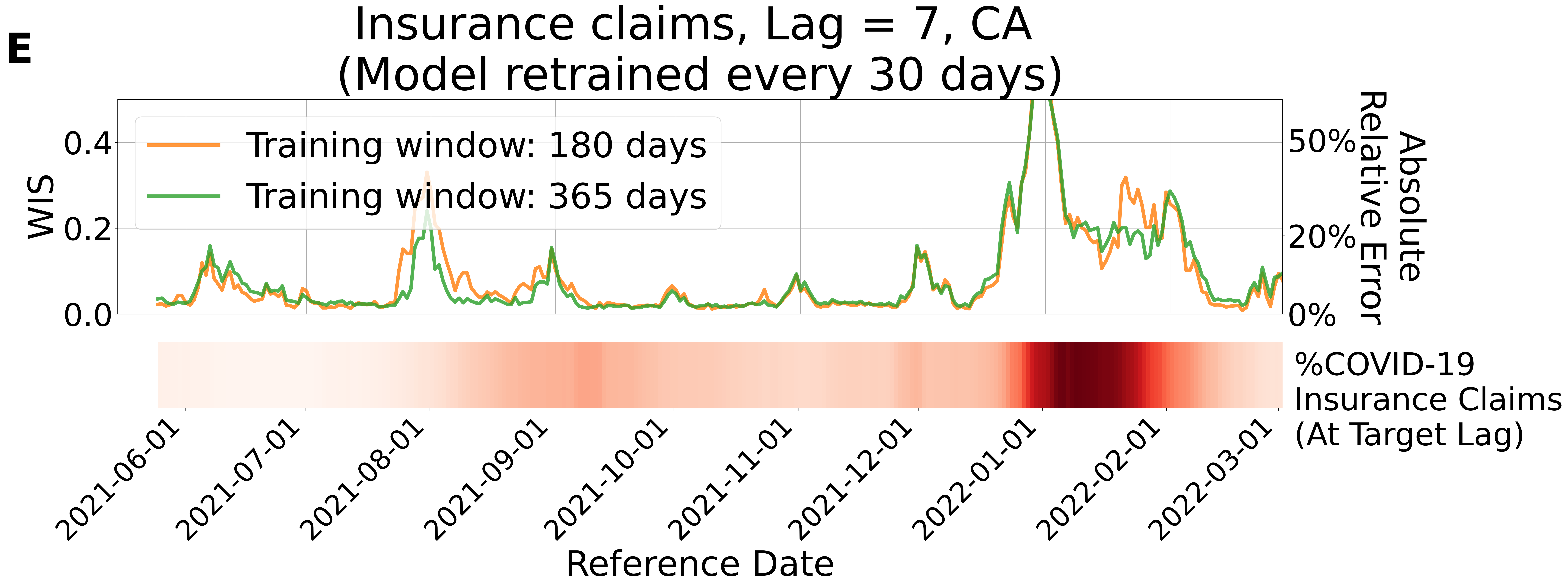
E
Insurance claims, Lag = 7, CA
(Model retrained every 30 days)
Training window: 180 days
Training window: 365 days
WIS
0.0
0.2
0.4
Absolute Relative Error
0%
20%
50%
%COVID-19 Insurance Claims (At Target Lag)
2021-06-01
2021-07-01
2021-08-01
2021-09-01
2021-10-01
2021-11-01
2021-12-01
2022-01-01
2022-02-01
2022-03-01
Reference Date

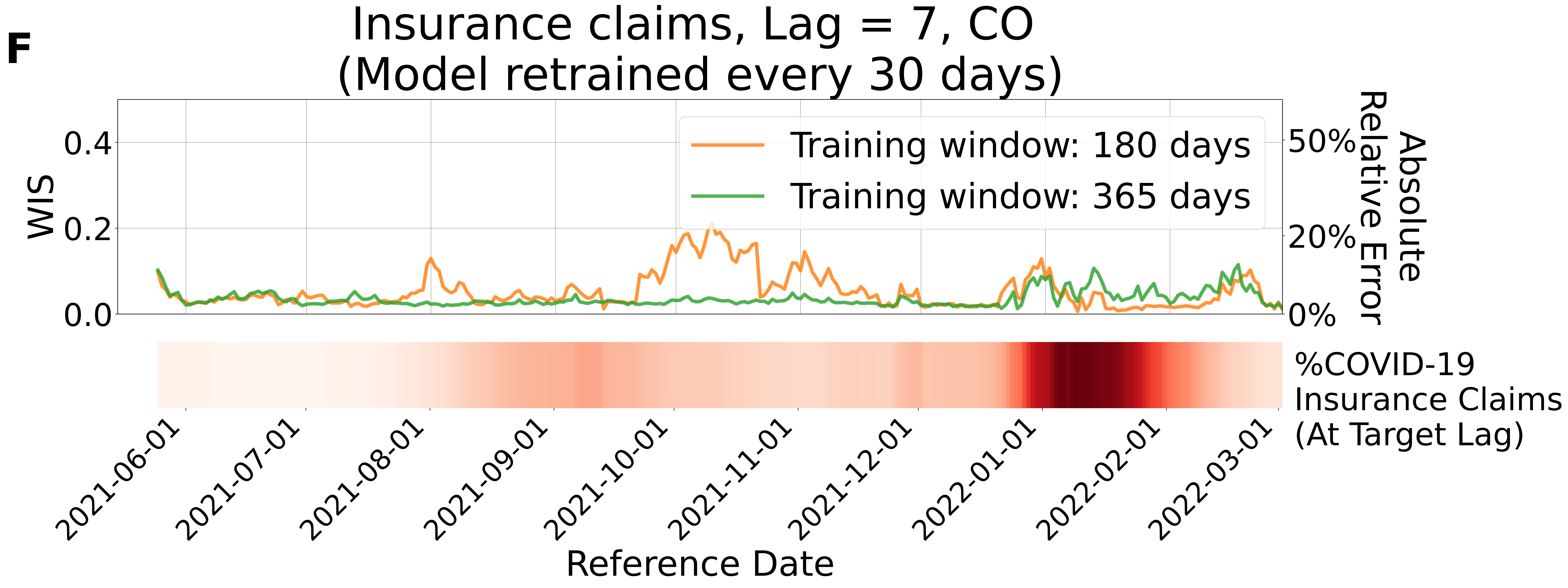
F
Insurance claims, Lag = 7, CO
(Model retrained every 30 days)
WIS
0.0
0.2
0.4
Absolute
Relative Error
0%
20%
50%
Training window: 180 days
Training window: 365 days
%COVID-19
Insurance Claims
(At Target Lag)
2021-06-01
2021-07-01
2021-08-01
2021-09-01
2021-10-01
2021-11-01
2021-12-01
2022-01-01
2022-02-01
2022-03-01
Reference Date

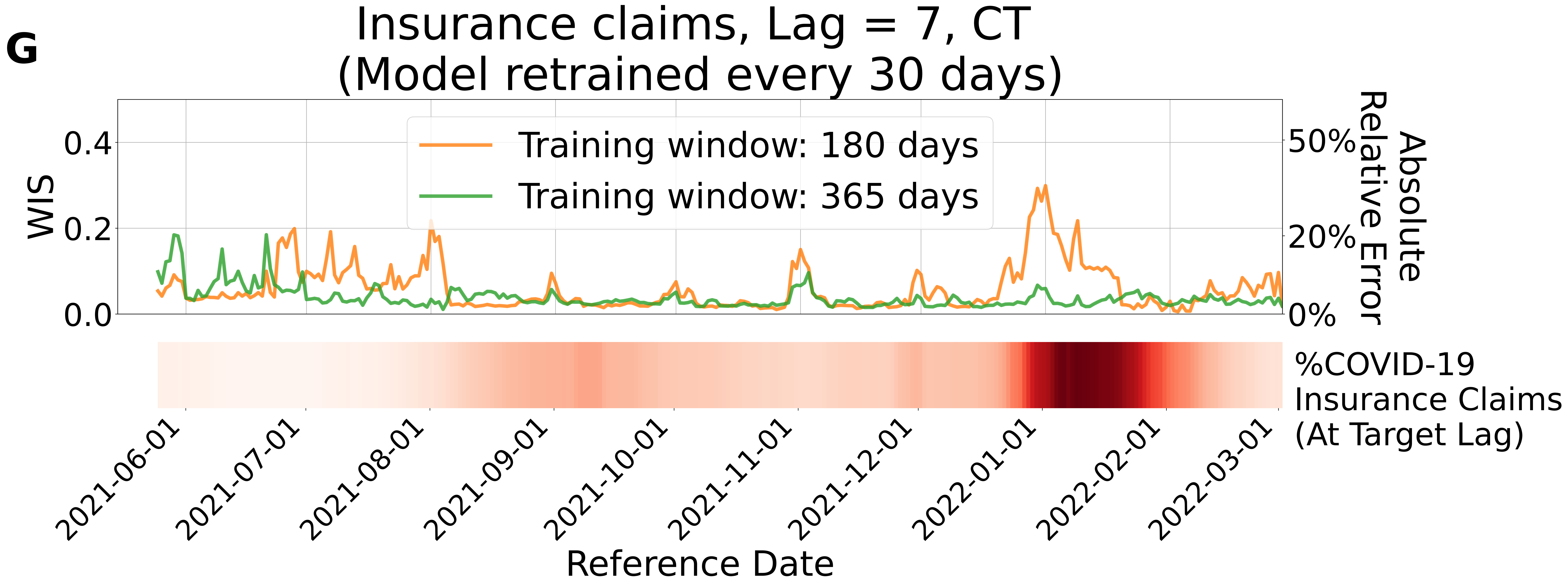

G
Insurance claims, Lag = 7, CT
(Model retrained every 30 days)
Training window: 180 days
Training window: 365 days
WIS
0.0
0.2
0.4
Absolute Relative Error
0%
20%
50%
%COVID-19 Insurance Claims (At Target Lag)
2021-06-01
2021-07-01
2021-08-01
2021-09-01
2021-10-01
2021-11-01
2021-12-01
2022-01-01
2022-02-01
2022-03-01
Reference Date

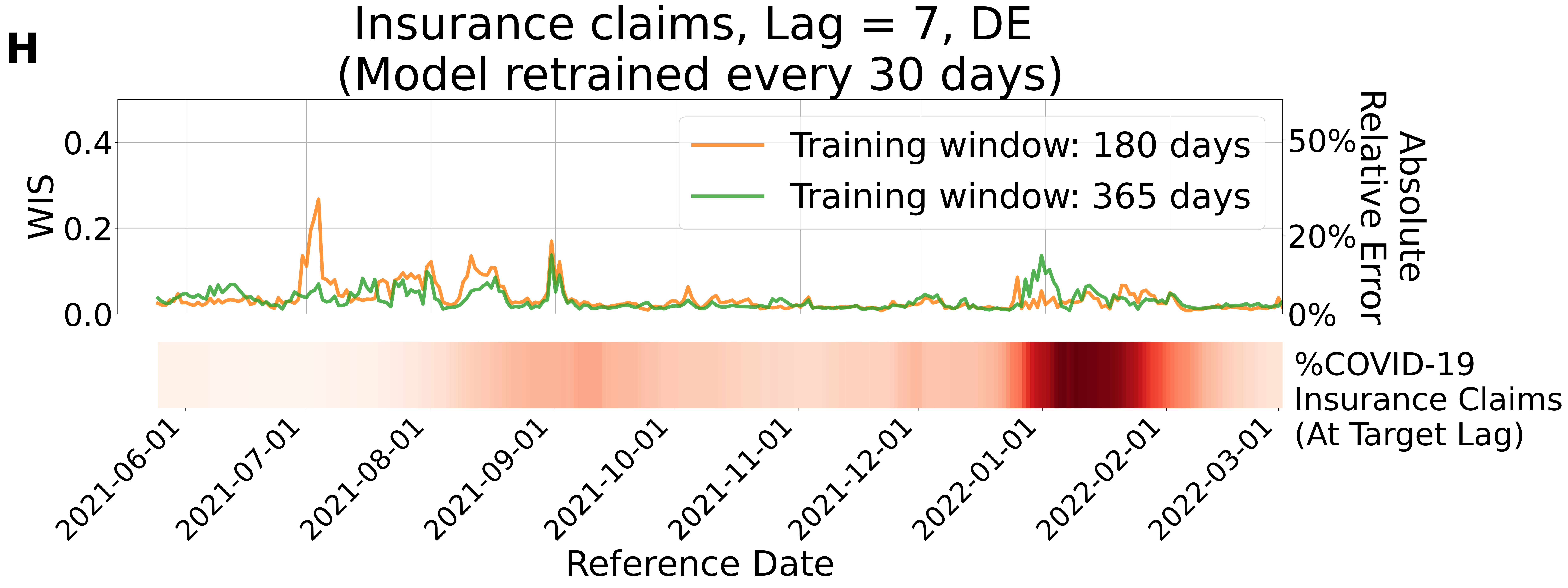
H
Insurance claims, Lag = 7, DE
(Model retrained every 30 days)
WIS
0.0
0.2
0.4
Absolute Relative Error
0%
20%
50%
Training window: 180 days
Training window: 365 days
%COVID-19 Insurance Claims (At Target Lag)
2021-06-01
2021-07-01
2021-08-01
2021-09-01
2021-10-01
2021-11-01
2021-12-01
2022-01-01
2022-02-01
2022-03-01
Reference Date

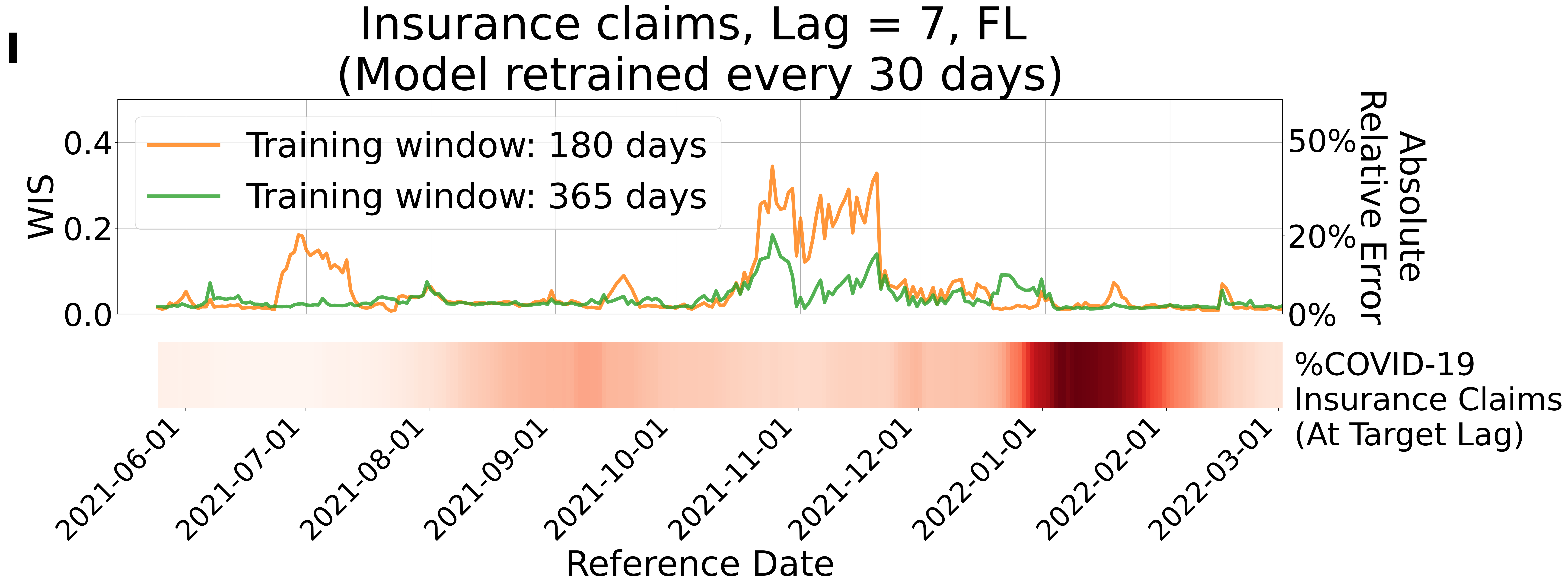
I
Insurance claims, Lag = 7, FL
(Model retrained every 30 days)
Training window: 180 days
Training window: 365 days
WIS
0.0
0.2
0.4
Absolute Relative Error
0%
20%
50%
%COVID-19 Insurance Claims (At Target Lag)
2021-06-01
2021-07-01
2021-08-01
2021-09-01
2021-10-01
2021-11-01
2021-12-01
2022-01-01
2022-02-01
2022-03-01
Reference Date

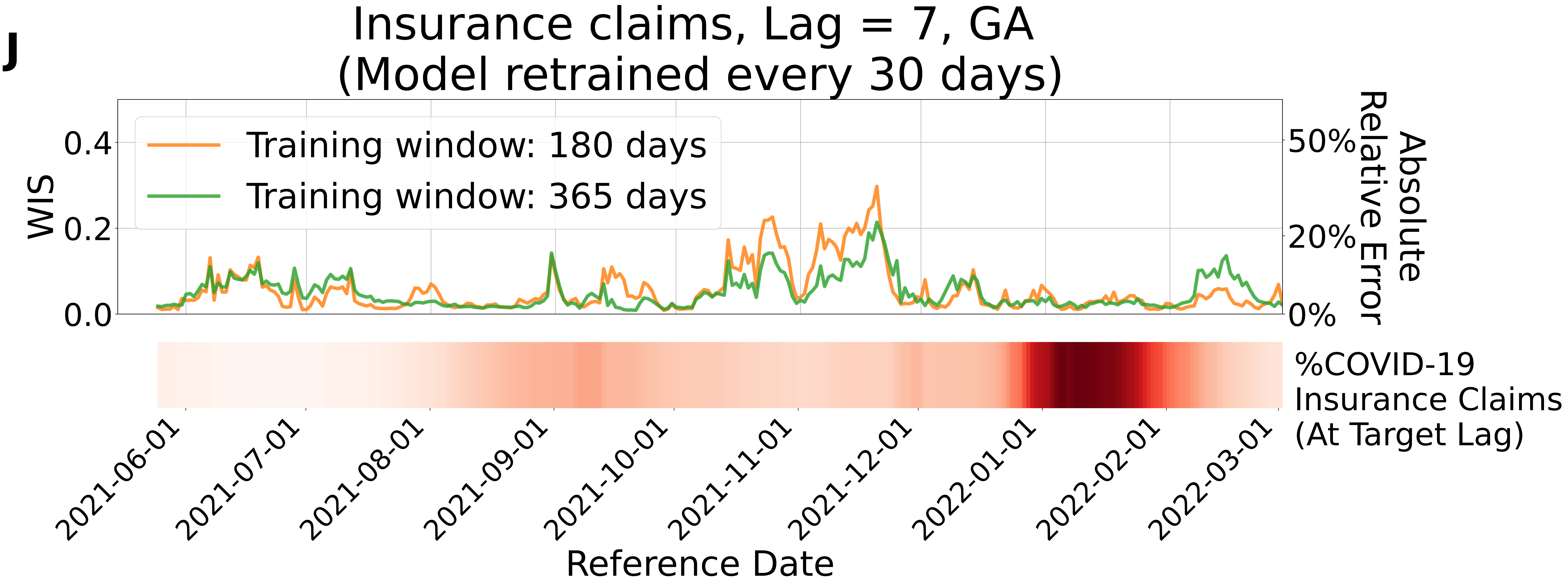

J
Insurance claims, Lag = 7, GA
(Model retrained every 30 days)
Training window: 180 days
Training window: 365 days
WIS
0.0
0.2
0.4
Absolute
Relative Error
0%
20%
50%
%COVID-19
Insurance Claims
(At Target Lag)
2021-06-01
2021-07-01
2021-08-01
2021-09-01
2021-10-01
2021-11-01
2021-12-01
2022-01-01
2022-02-01
2022-03-01
Reference Date

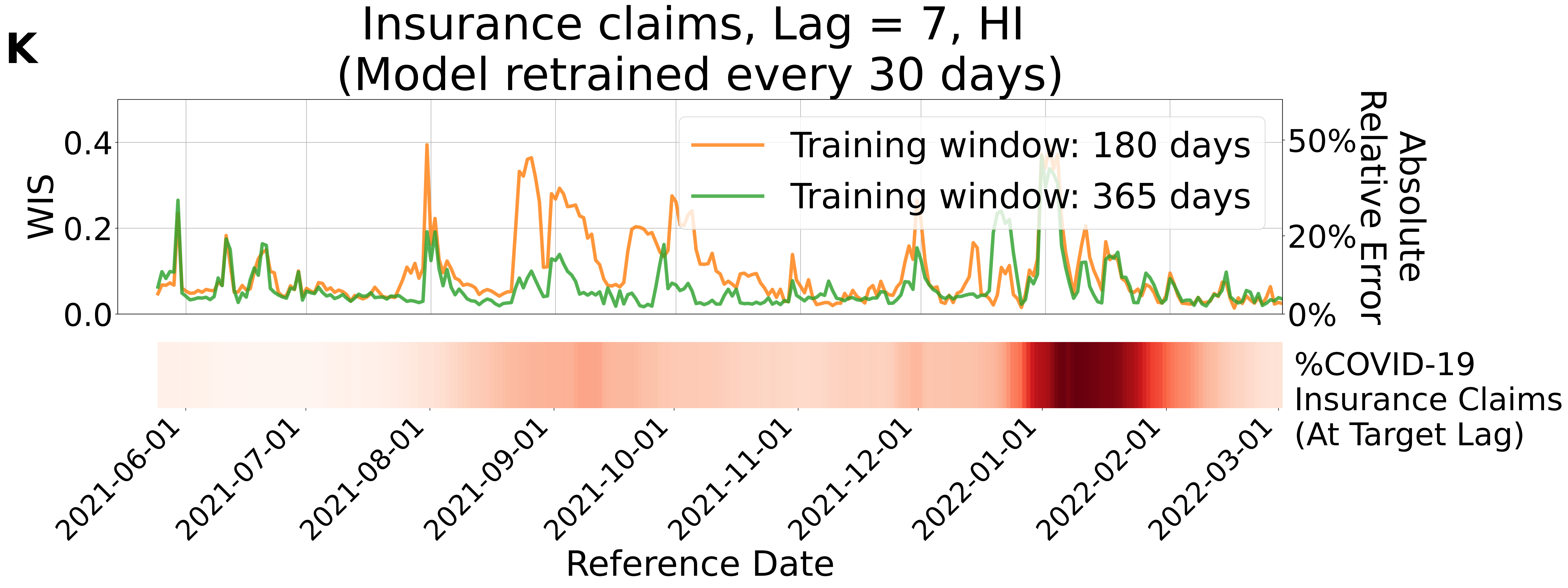
K
Insurance claims, Lag = 7, HI
(Model retrained every 30 days)
WIS
0.0
0.2
0.4
Absolute Relative Error
0%
20%
50%
Training window: 180 days
Training window: 365 days
%COVID-19
Insurance Claims
(At Target Lag)
2021-06-01
2021-07-01
2021-08-01
2021-09-01
2021-10-01
2021-11-01
2021-12-01
2022-01-01
2022-02-01
2022-03-01
Reference Date

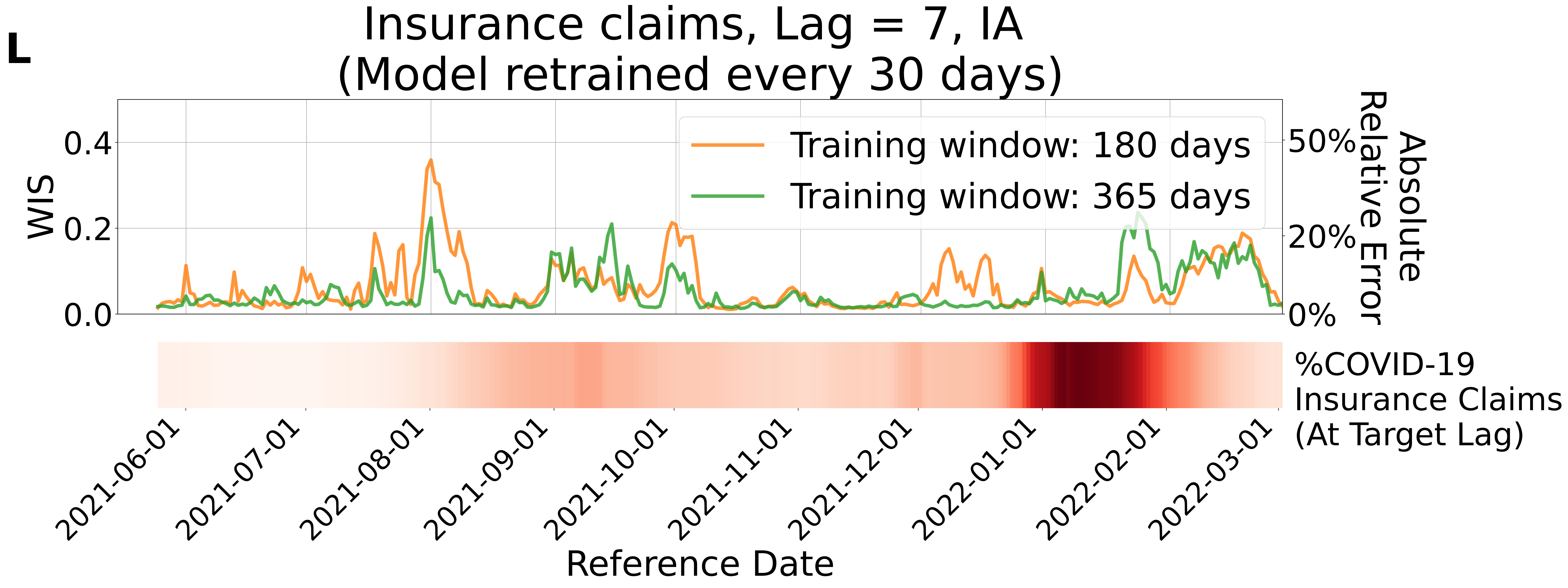

L
Insurance claims, Lag = 7, IA
(Model retrained every 30 days)
WIS
0.0
0.2
0.4
Absolute Relative Error
0%
20%
50%
Training window: 180 days
Training window: 365 days
%COVID-19 Insurance Claims (At Target Lag)
2021-06-01
2021-07-01
2021-08-01
2021-09-01
2021-10-01
2021-11-01
2021-12-01
2022-01-01
2022-02-01
2022-03-01
Reference Date

M

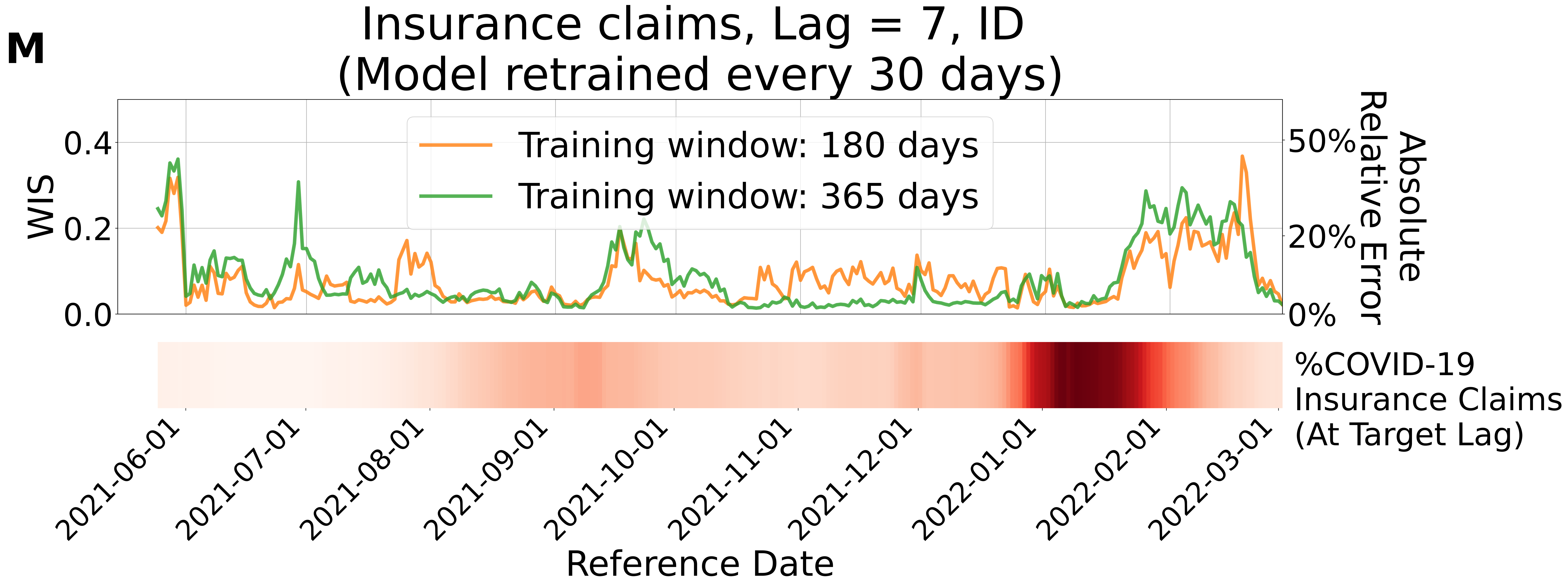

Insurance claims, Lag = 7, ID
(Model retrained every 30 days)
WIS
0.4
0.2
0.0
Training window: 180 days
Training window: 365 days
Absolute Relative Error
50%
20%
0%
%COVID-19 Insurance Claims (At Target Lag)
2021-06-01
2021-07-01
2021-08-01
2021-09-01
2021-10-01
2021-11-01
2021-12-01
2022-01-01
2022-02-01
2022-03-01
Reference Date

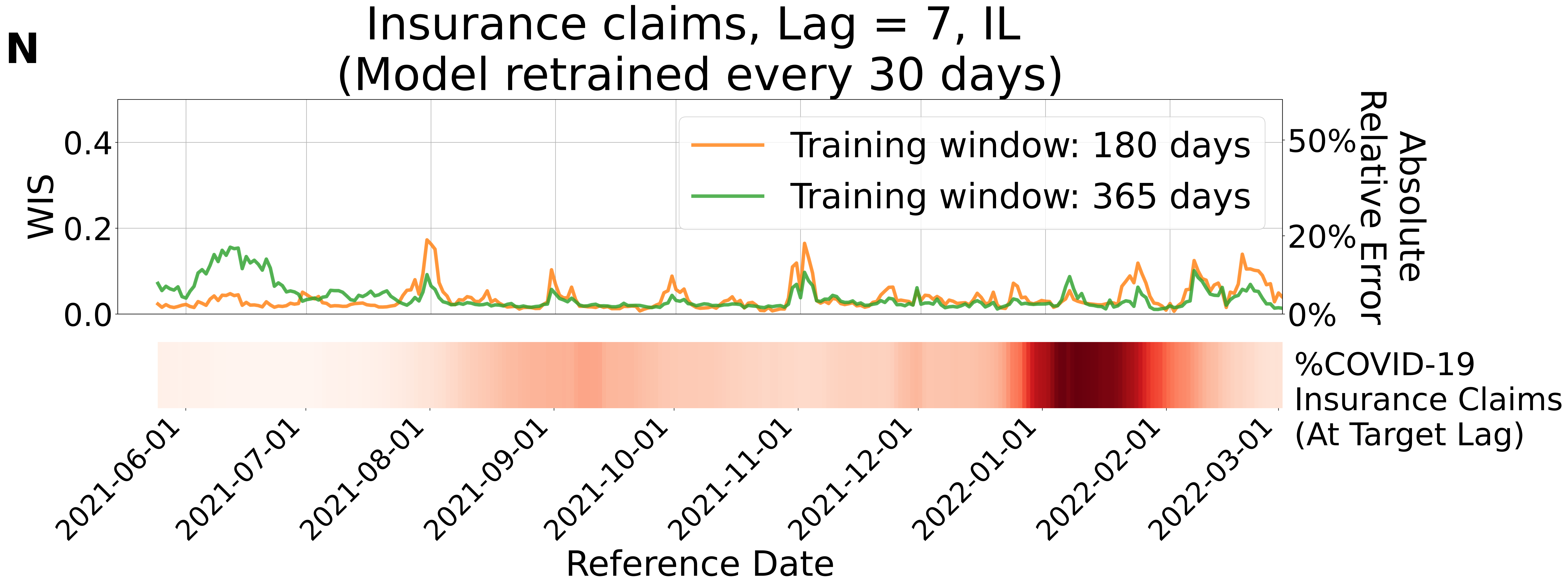
N
Insurance claims, Lag = 7, IL
(Model retrained every 30 days)
WIS
0.4
0.2
0.0
Absolute Relative Error
50%
20%
0%
Training window: 180 days
Training window: 365 days
%COVID-19 Insurance Claims (At Target Lag)
2021-06-01
2021-07-01
2021-08-01
2021-09-01
2021-10-01
2021-11-01
2021-12-01
2022-01-01
2022-02-01
2022-03-01
Reference Date

O

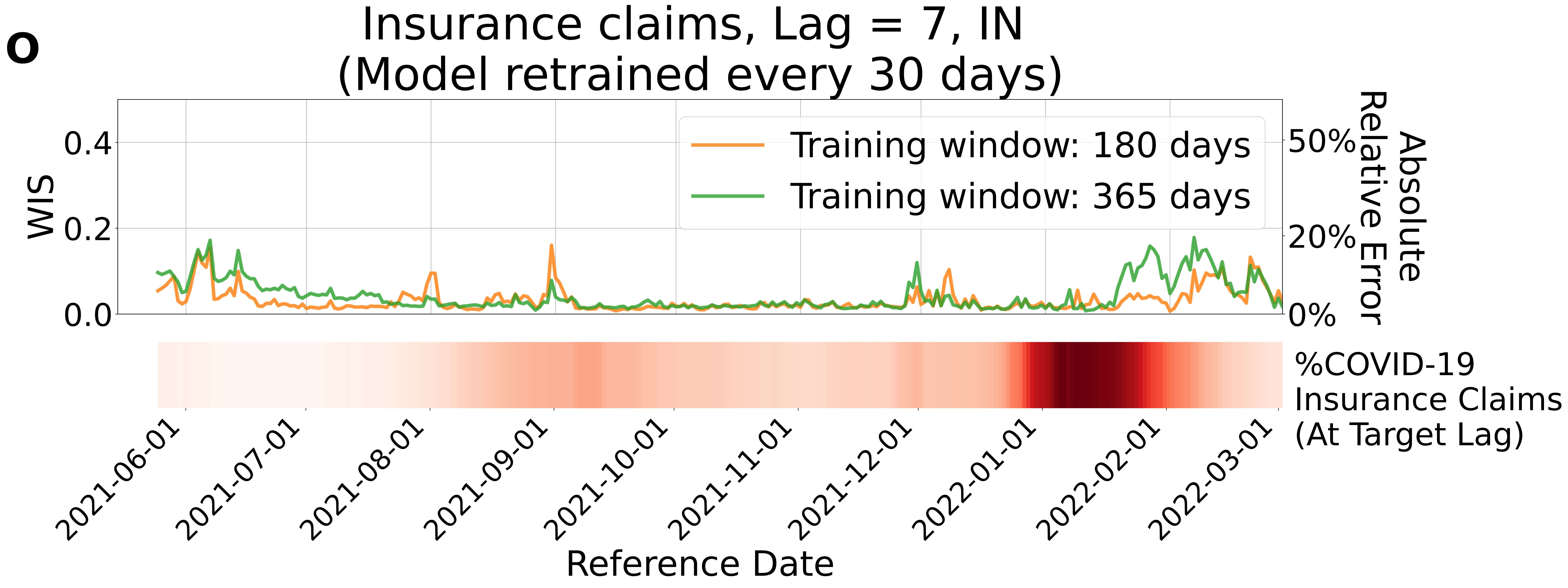
Insurance claims, Lag = 7, IN
(Model retrained every 30 days)
WIS
0.4
0.2
0.0
Absolute Relative Error
50%
20%
0%
Training window: 180 days
Training window: 365 days
%COVID-19
Insurance Claims
(At Target Lag)
2021-06-01
2021-07-01
2021-08-01
2021-09-01
2021-10-01
2021-11-01
2021-12-01
2022-01-01
2022-02-01
2022-03-01
Reference Date

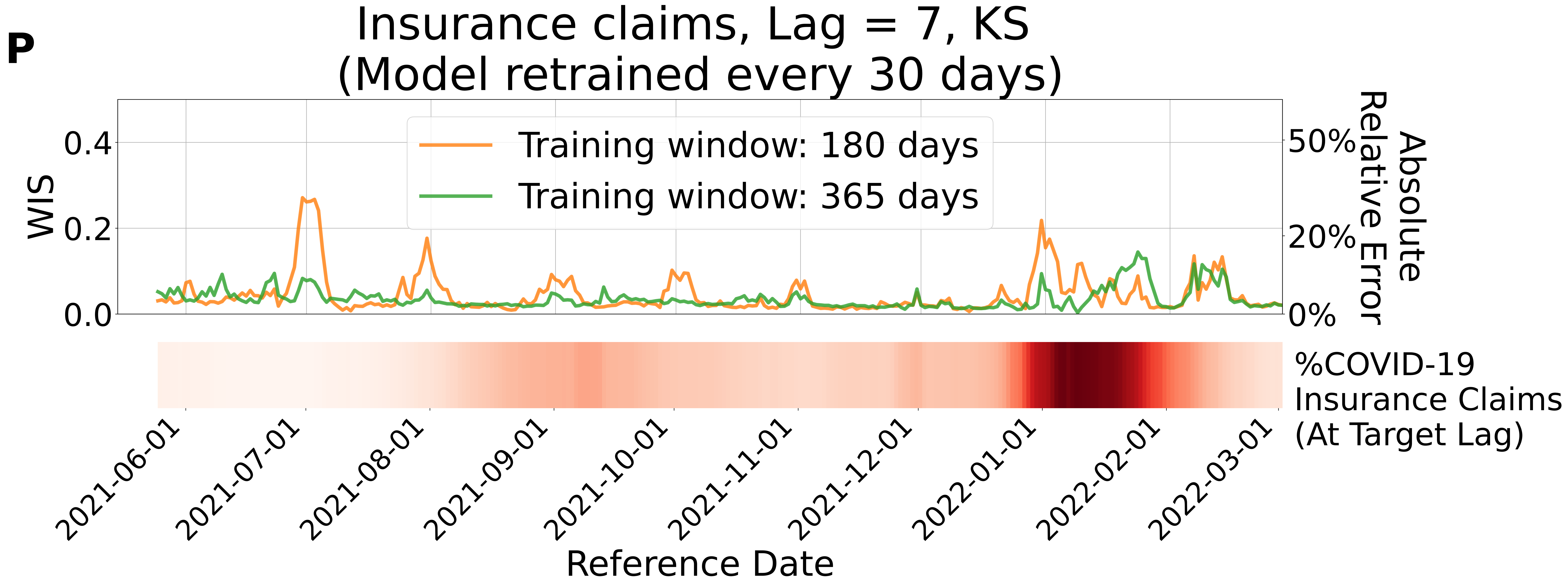
P
Insurance claims, Lag = 7, KS
(Model retrained every 30 days)
Training window: 180 days
Training window: 365 days
WIS
0.0
0.2
0.4
Absolute Relative Error
0%
20%
50%
%COVID-19 Insurance Claims (At Target Lag)
2021-06-01
2021-07-01
2021-08-01
2021-09-01
2021-10-01
2021-11-01
2021-12-01
2022-01-01
2022-02-01
2022-03-01
Reference Date

Q

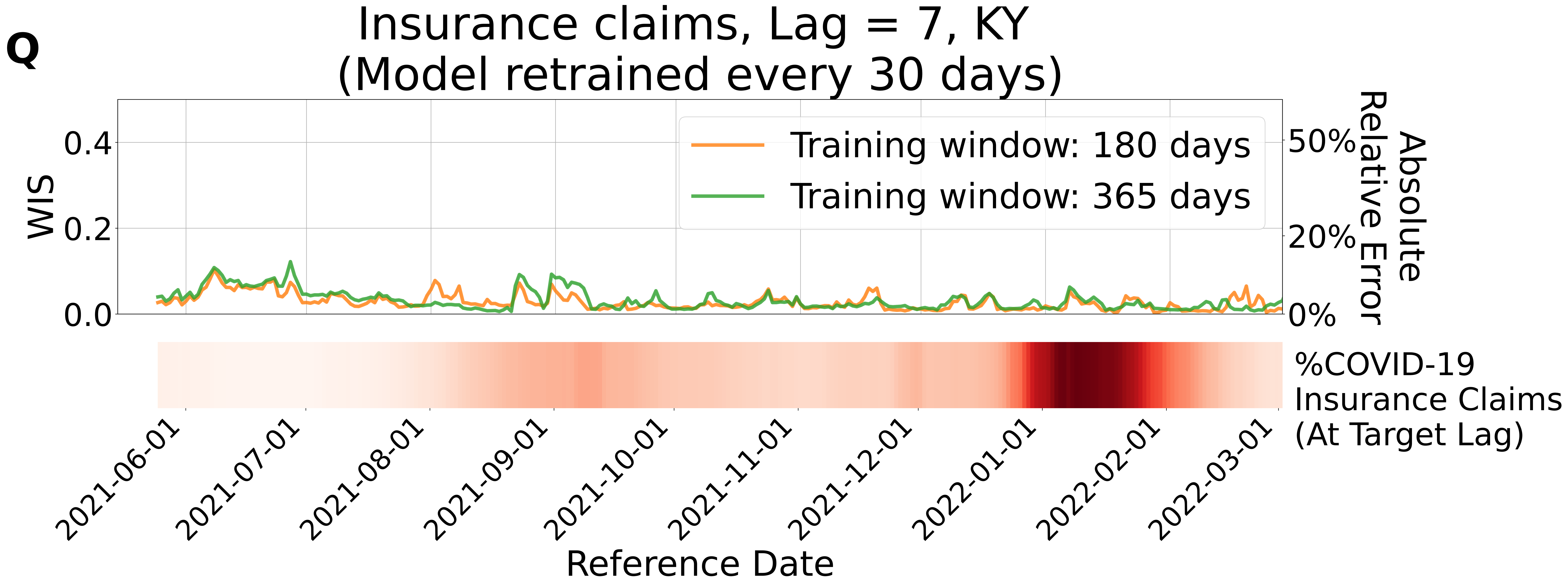

Insurance claims, Lag = 7, KY
(Model retrained every 30 days)
WIS
0.4
0.2
0.0
Absolute Relative Error
50%
20%
0%
Training window: 180 days
Training window: 365 days
%COVID-19 Insurance Claims (At Target Lag)
2021-06-01
2021-07-01
2021-08-01
2021-09-01
2021-10-01
2021-11-01
2021-12-01
2022-01-01
2022-02-01
2022-03-01
Reference Date

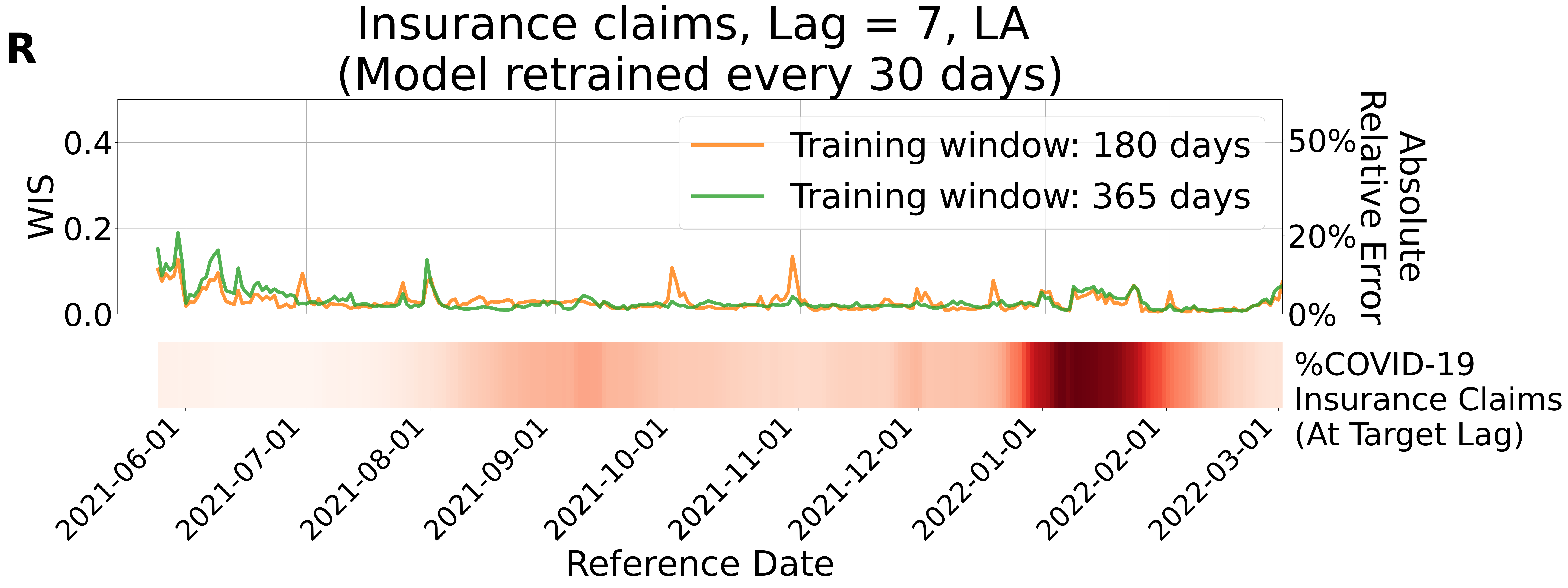
R
Insurance claims, Lag = 7, LA
(Model retrained every 30 days)
WIS
0.0
0.2
0.4
Absolute Relative Error
0%
20%
50%
Training window: 180 days
Training window: 365 days
%COVID-19 Insurance Claims (At Target Lag)
2021-06-01
2021-07-01
2021-08-01
2021-09-01
2021-10-01
2021-11-01
2021-12-01
2022-01-01
2022-02-01
2022-03-01
Reference Date

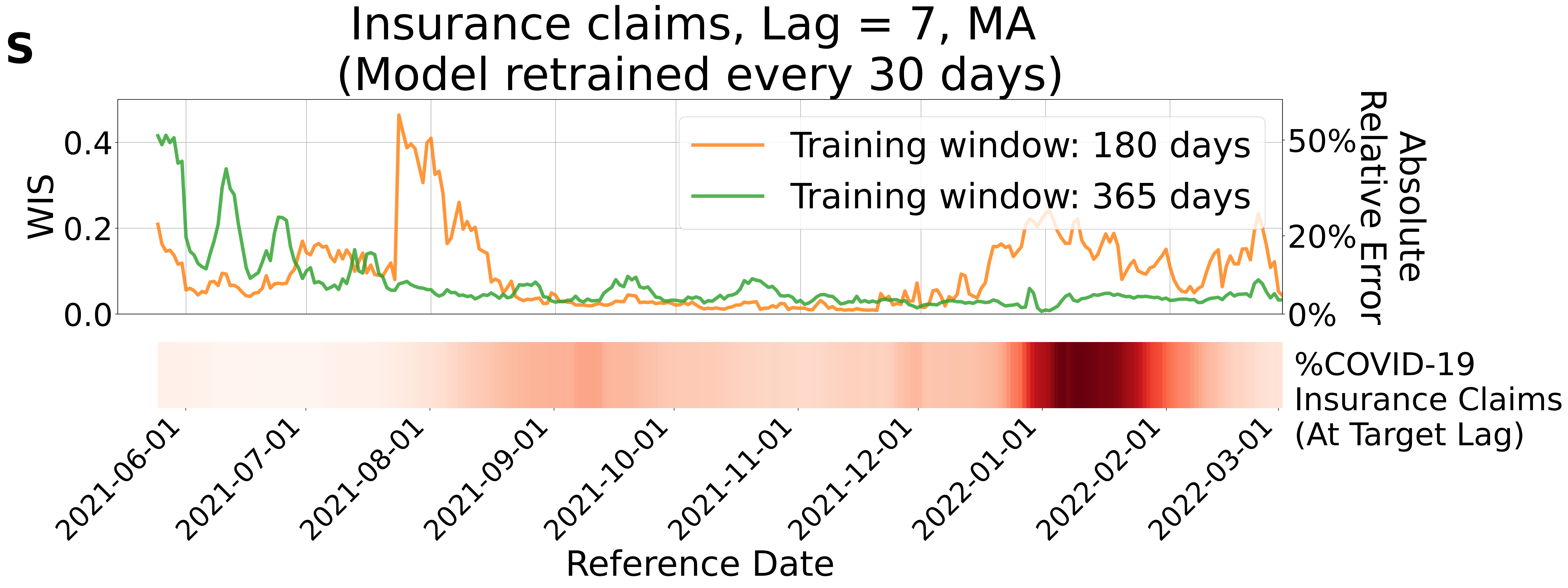

s
Insurance claims, Lag = 7, MA
(Model retrained every 30 days)
Training window: 180 days
Training window: 365 days
WIS
0.0
0.2
0.4
Absolute Relative Error
0%
20%
50%
%COVID-19 Insurance Claims (At Target Lag)
2021-06-01
2021-07-01
2021-08-01
2021-09-01
2021-10-01
2021-11-01
2021-12-01
2022-01-01
2022-02-01
2022-03-01
Reference Date

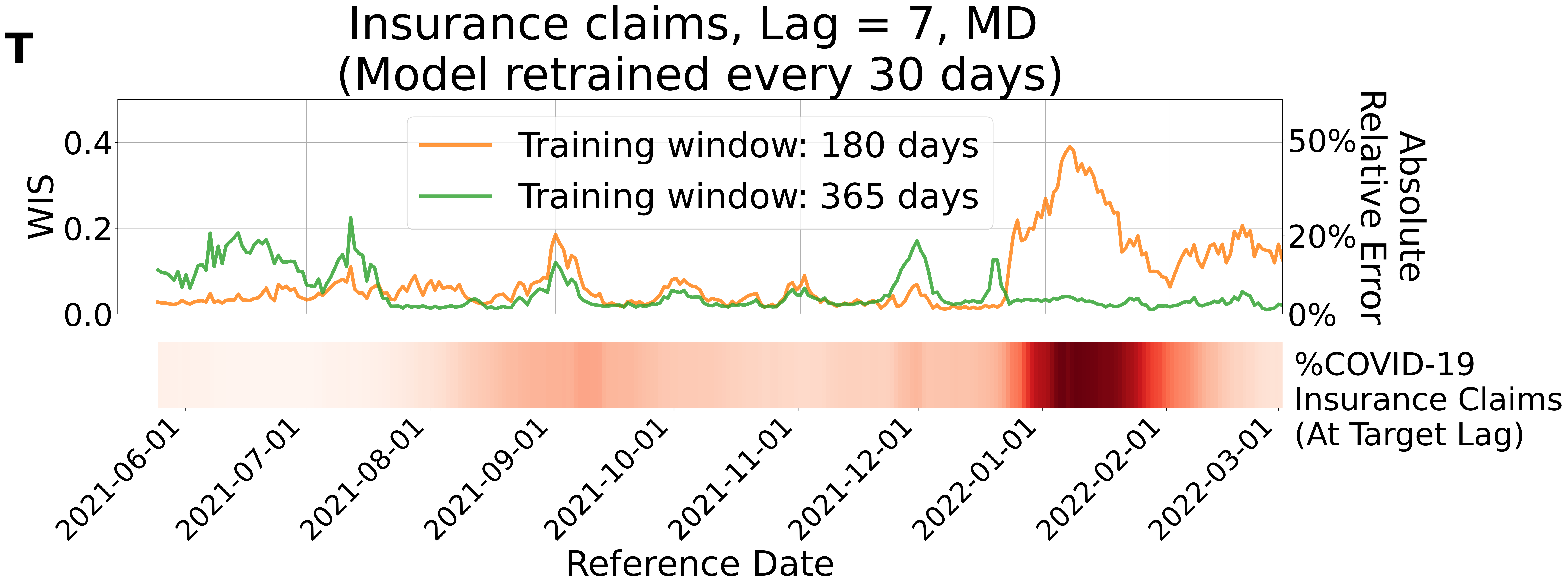
T
Insurance claims, Lag = 7, MD
(Model retrained every 30 days)
Training window: 180 days
Training window: 365 days
WIS
0.0
0.2
0.4
0%
20%
50%
Absolute Relative Error
%COVID-19 Insurance Claims (At Target Lag)
2021-06-01
2021-07-01
2021-08-01
2021-09-01
2021-10-01
2021-11-01
2021-12-01
2022-01-01
2022-02-01
2022-03-01
Reference Date

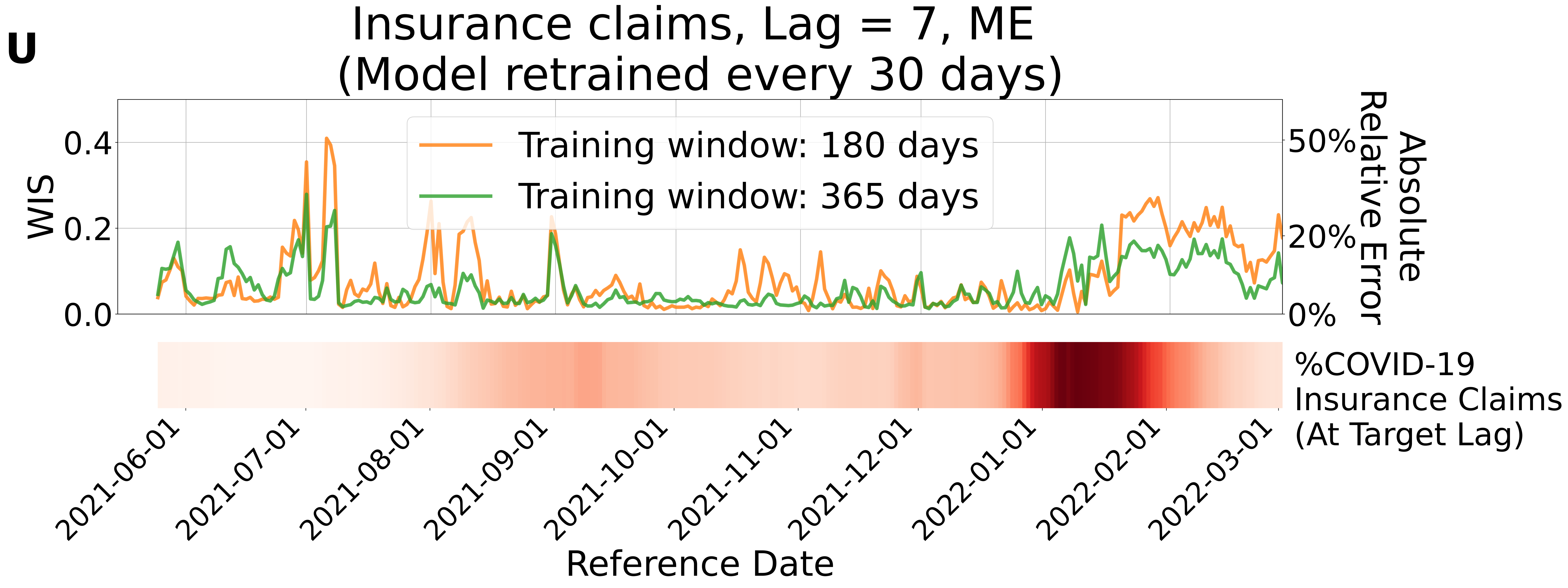
U
Insurance claims, Lag = 7, ME
(Model retrained every 30 days)
Training window: 180 days
Training window: 365 days
WIS
0.0
0.2
0.4
Absolute Relative Error
0%
20%
50%
%COVID-19 Insurance Claims (At Target Lag)
2021-06-01
2021-07-01
2021-08-01
2021-09-01
2021-10-01
2021-11-01
2021-12-01
2022-01-01
2022-02-01
2022-03-01
Reference Date

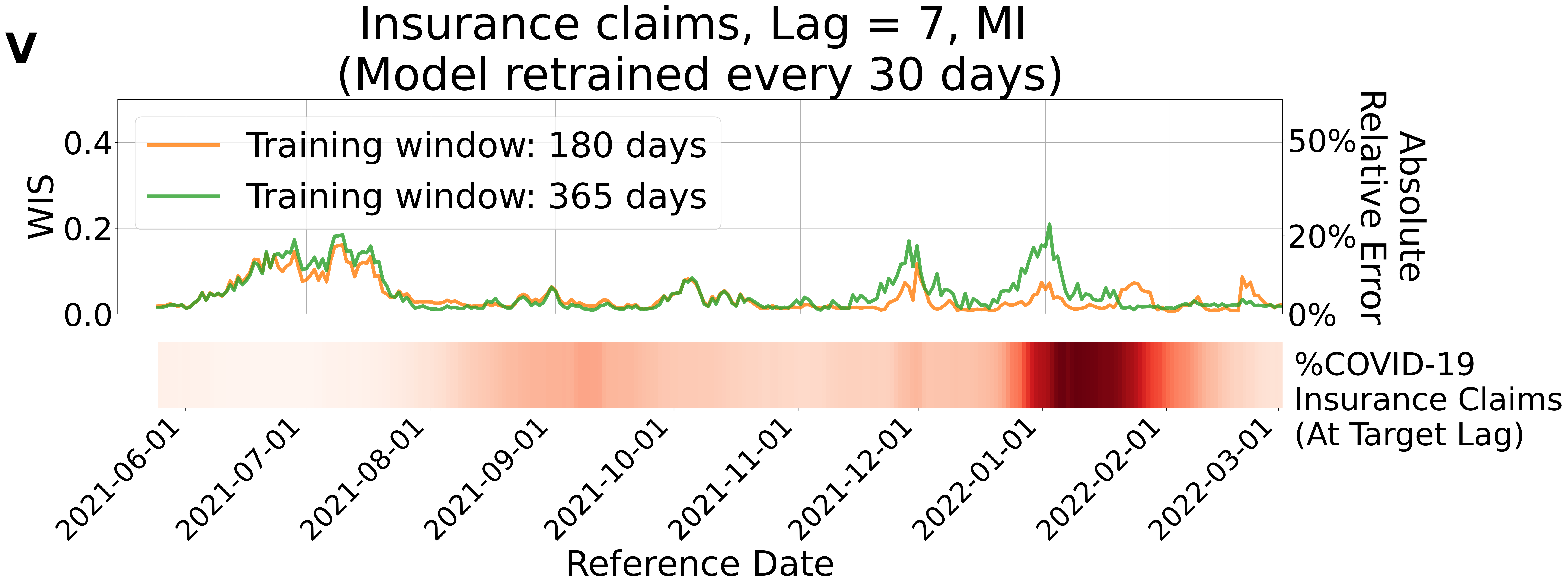
v
Insurance claims, Lag = 7, MI
(Model retrained every 30 days)
Training window: 180 days
Training window: 365 days
WIS
0.0
0.2
0.4
Absolute Relative Error
0%
20%
50%
%COVID-19 Insurance Claims (At Target Lag)
2021-06-01
2021-07-01
2021-08-01
2021-09-01
2021-10-01
2021-11-01
2021-12-01
2022-01-01
2022-02-01
2022-03-01
Reference Date

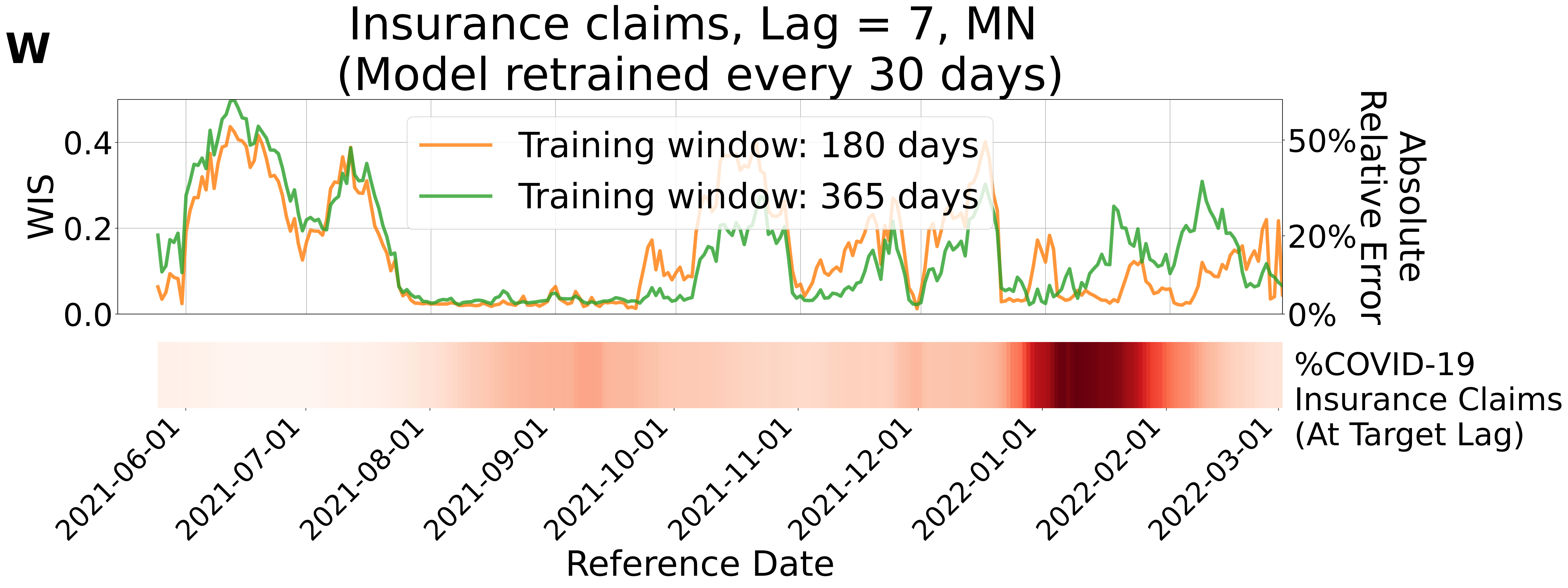

w
Insurance claims, Lag = 7, MN
(Model retrained every 30 days)
Training window: 180 days
Training window: 365 days
WIS
0.0
0.2
0.4
Absolute Relative Error
0%
20%
50%
%COVID-19 Insurance Claims (At Target Lag)
2021-06-01
2021-07-01
2021-08-01
2021-09-01
2021-10-01
2021-11-01
2021-12-01
2022-01-01
2022-02-01
2022-03-01
Reference Date

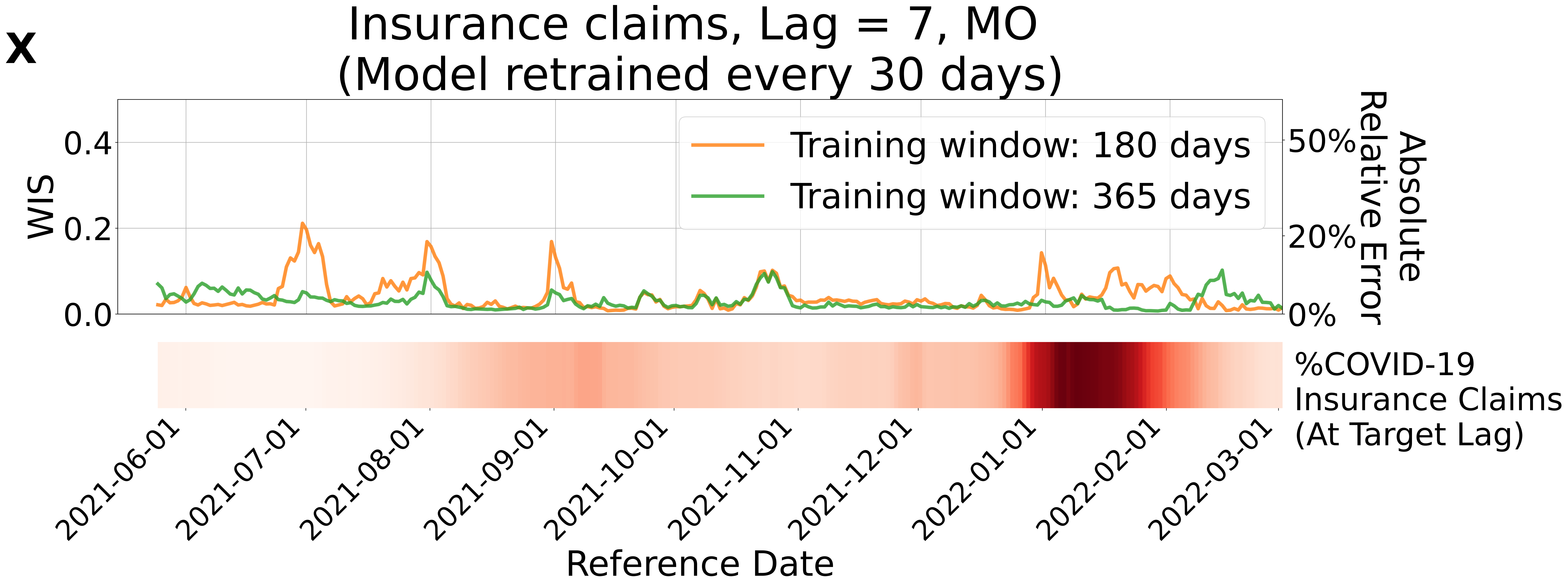
X
Insurance claims, Lag = 7, MO
(Model retrained every 30 days)
WIS
0.4
0.2
0.0
Absolute Relative Error
50%
20%
0%
Training window: 180 days
Training window: 365 days
%COVID-19 Insurance Claims (At Target Lag)
2021-06-01
2021-07-01
2021-08-01
2021-09-01
2021-10-01
2021-11-01
2021-12-01
2022-01-01
2022-02-01
2022-03-01
Reference Date

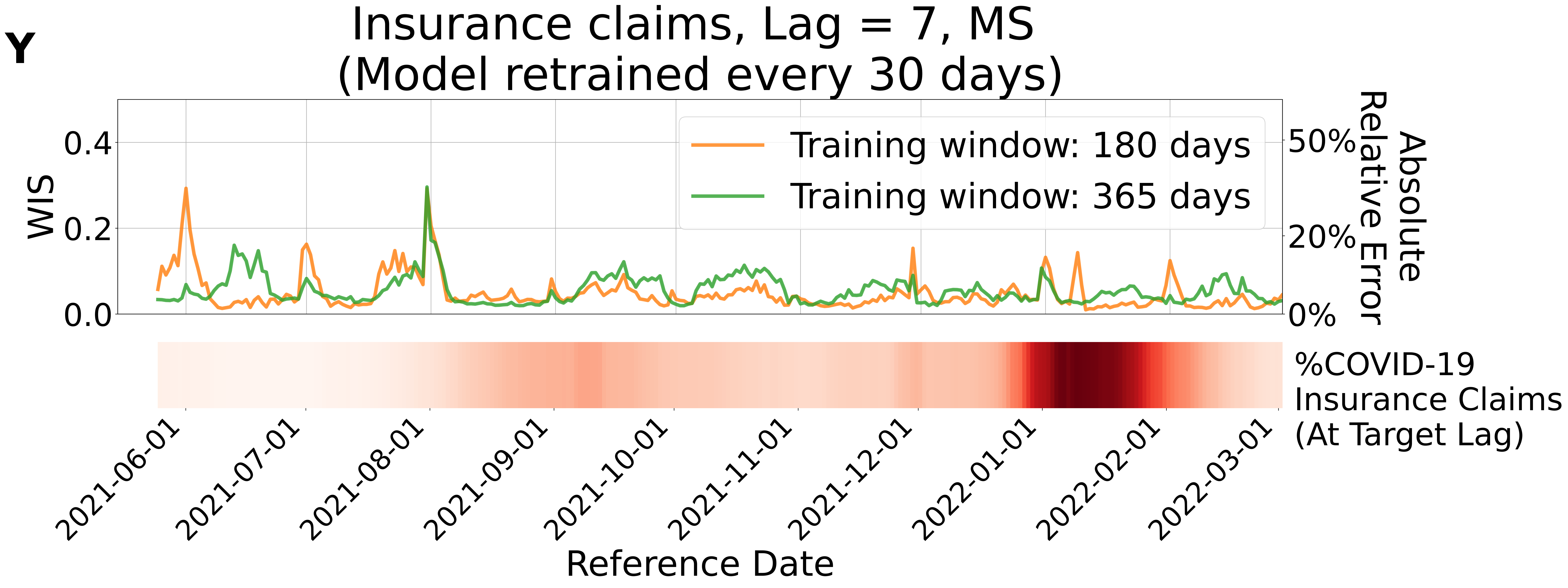
Y
Insurance claims, Lag = 7, MS
(Model retrained every 30 days)
WIS
0.4
0.2
0.0
50%
20%
0%
Absolute
Relative Error
Training window: 180 days
Training window: 365 days
%COVID-19
Insurance Claims
(At Target Lag)
2021-06-01
2021-07-01
2021-08-01
2021-09-01
2021-10-01
2021-11-01
2021-12-01
2022-01-01
2022-02-01
2022-03-01
Reference Date

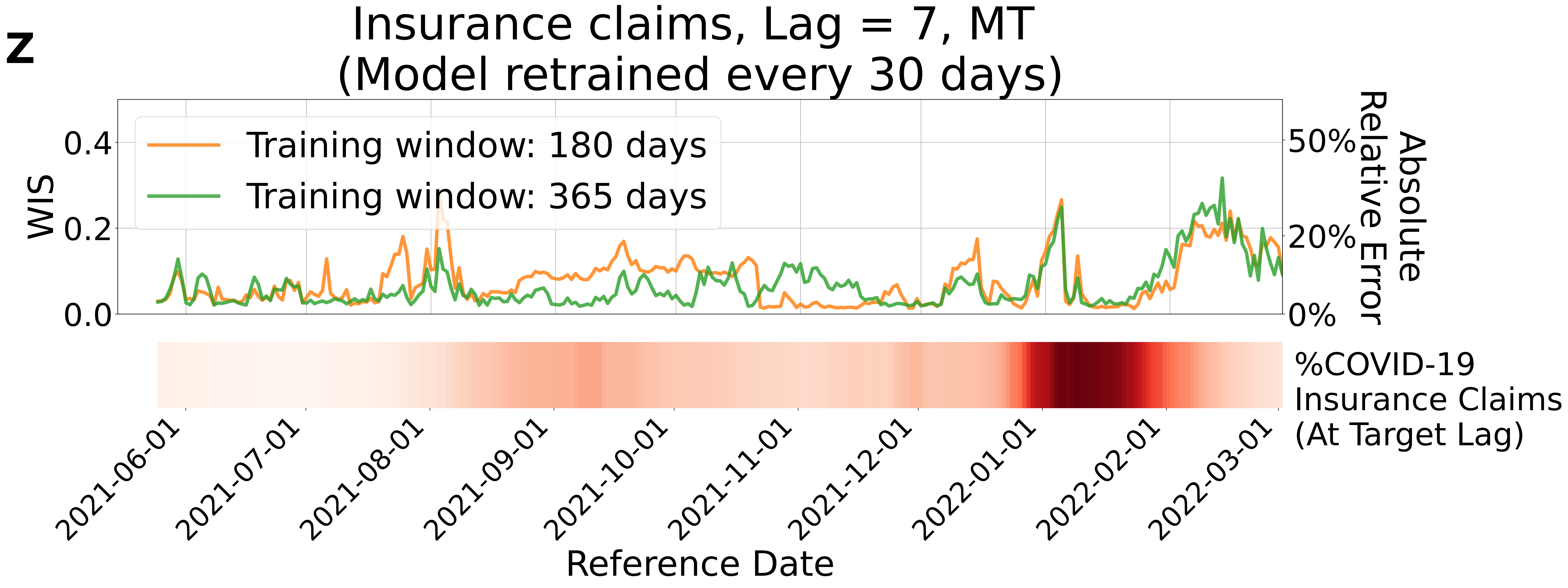
z
Insurance claims, Lag = 7, MT
(Model retrained every 30 days)
Training window: 180 days
Training window: 365 days
WIS
0.0
0.2
0.4
Absolute
Relative Error
0%
20%
50%
%COVID-19
Insurance Claims
(At Target Lag)
2021-06-01
2021-07-01
2021-08-01
2021-09-01
2021-10-01
2021-11-01
2021-12-01
2022-01-01
2022-02-01
2022-03-01
Reference Date

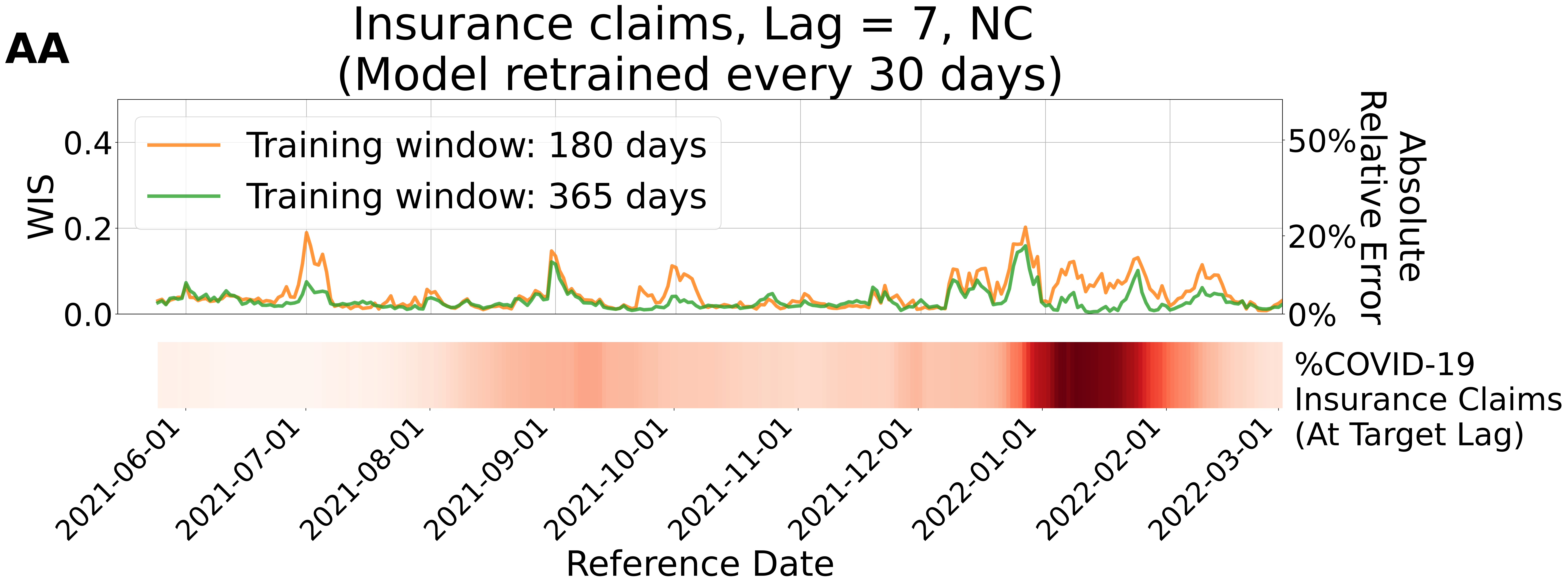
AA
Insurance claims, Lag = 7, NC
(Model retrained every 30 days)
Training window: 180 days
Training window: 365 days
WIS
0.0
0.2
0.4
Absolute Relative Error
0%
20%
50%
%COVID-19
Insurance Claims
(At Target Lag)
2021-06-01
2021-07-01
2021-08-01
2021-09-01
2021-10-01
2021-11-01
2021-12-01
2022-01-01
2022-02-01
2022-03-01
Reference Date

AB

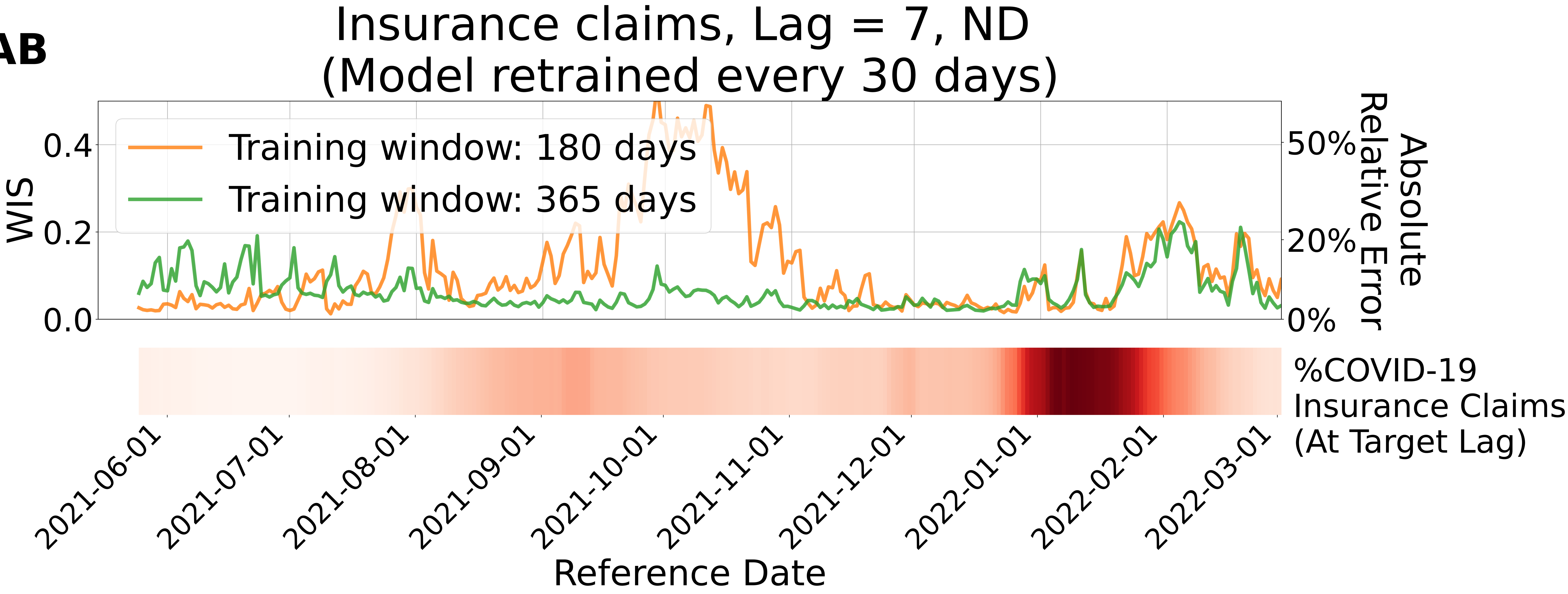

Insurance claims, Lag = 7, ND
(Model retrained every 30 days)
Training window: 180 days
Training window: 365 days
WIS
0.0
0.2
0.4
Absolute Relative Error
0%
20%
50%
%COVID-19 Insurance Claims (At Target Lag)
2021-06-01
2021-07-01
2021-08-01
2021-09-01
2021-10-01
2021-11-01
2021-12-01
2022-01-01
2022-02-01
2022-03-01
Reference Date

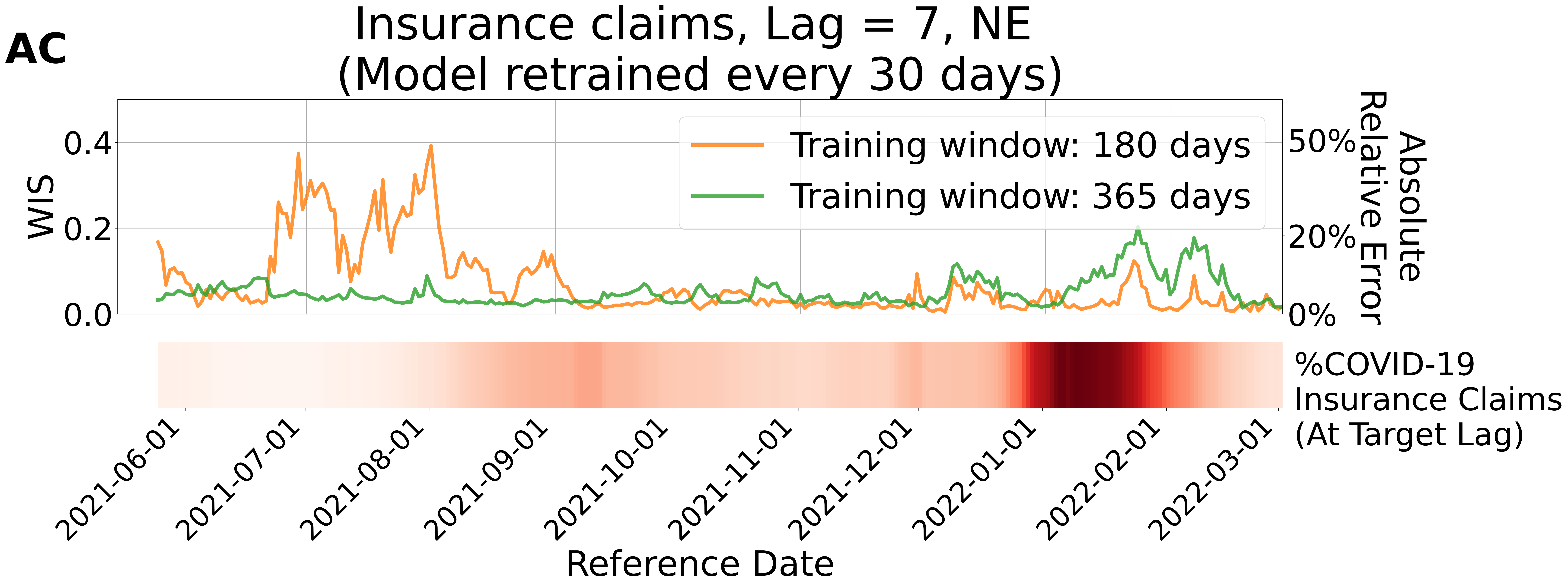
AC
Insurance claims, Lag = 7, NE
(Model retrained every 30 days)
WIS
0.0
0.2
0.4
Absolute Relative Error
0%
20%
50%
Training window: 180 days
Training window: 365 days
%COVID-19 Insurance Claims (At Target Lag)
2021-06-01
2021-07-01
2021-08-01
2021-09-01
2021-10-01
2021-11-01
2021-12-01
2022-01-01
2022-02-01
2022-03-01
Reference Date

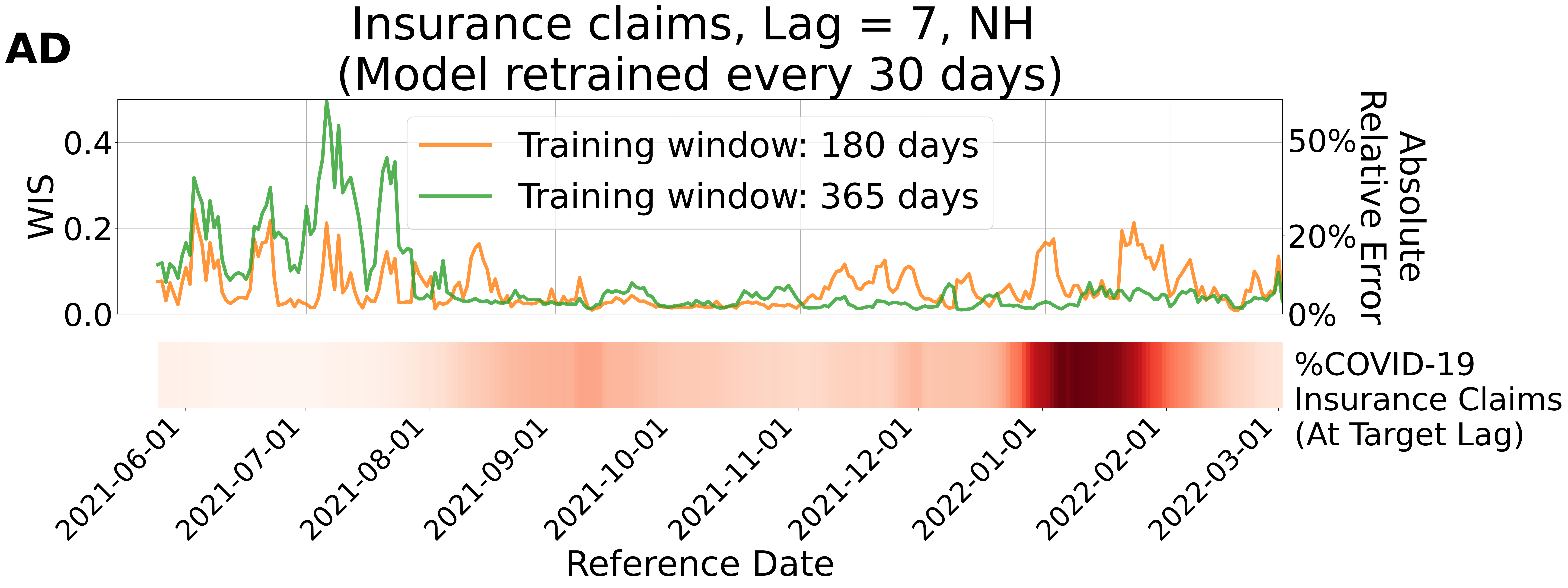

AD
Insurance claims, Lag = 7, NH
(Model retrained every 30 days)
WIS
0.0
0.2
0.4
Absolute Relative Error
0%
20%
50%
Training window: 180 days
Training window: 365 days
%COVID-19 Insurance Claims (At Target Lag)
2021-06-01
2021-07-01
2021-08-01
2021-09-01
2021-10-01
2021-11-01
2021-12-01
2022-01-01
2022-02-01
2022-03-01
Reference Date

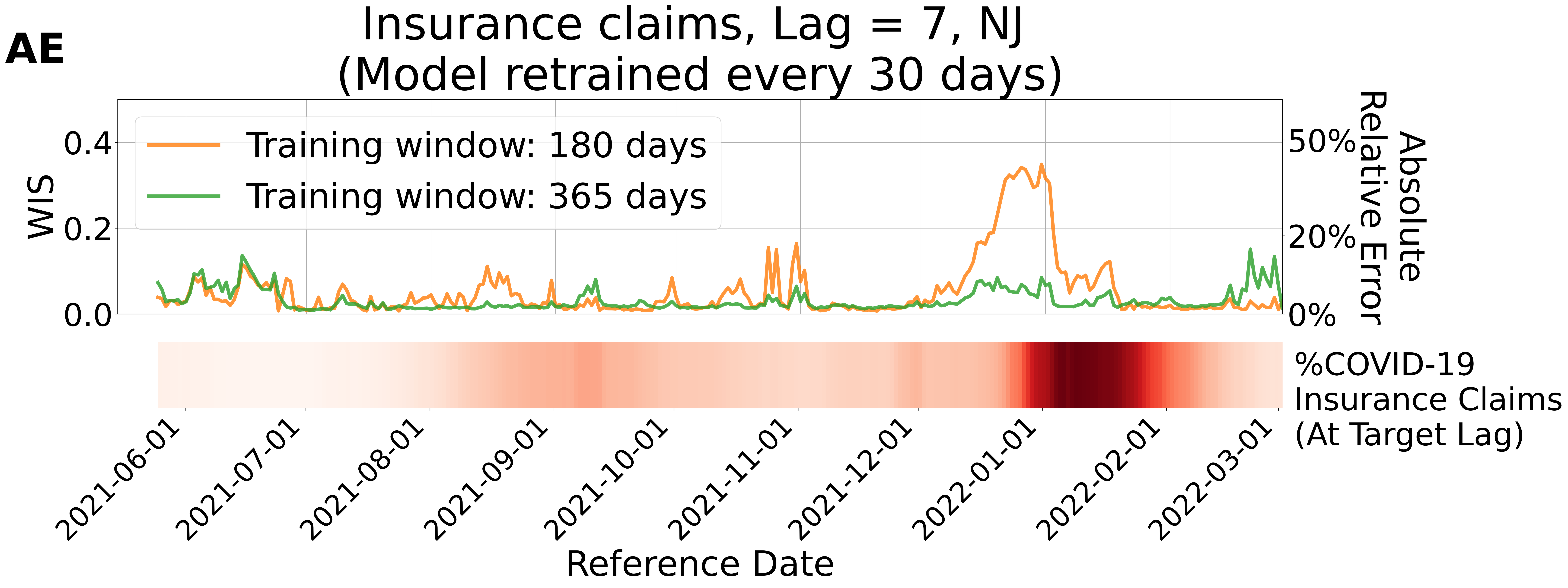

AE
Insurance claims, Lag = 7, NJ
(Model retrained every 30 days)
Training window: 180 days
Training window: 365 days
WIS
0.0
0.2
0.4
0%
20%
50%
Absolute
Relative Error
%COVID-19
Insurance Claims
(At Target Lag)
2021-06-01
2021-07-01
2021-08-01
2021-09-01
2021-10-01
2021-11-01
2021-12-01
2022-01-01
2022-02-01
2022-03-01
Reference Date

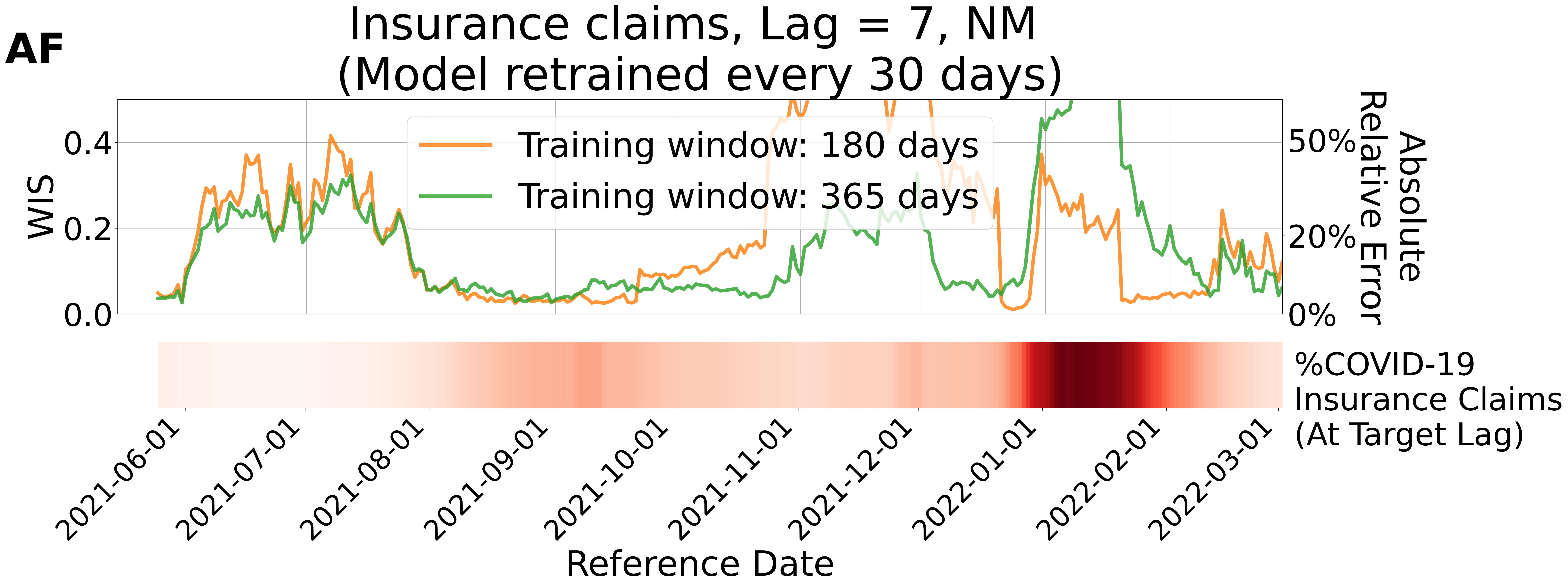
AF
Insurance claims, Lag = 7, NM
(Model retrained every 30 days)
Training window: 180 days
Training window: 365 days
WIS
0.0
0.2
0.4
Absolute Relative Error
0%
20%
50%
%COVID-19 Insurance Claims (At Target Lag)
2021-06-01
2021-07-01
2021-08-01
2021-09-01
2021-10-01
2021-11-01
2021-12-01
2022-01-01
2022-02-01
2022-03-01
Reference Date

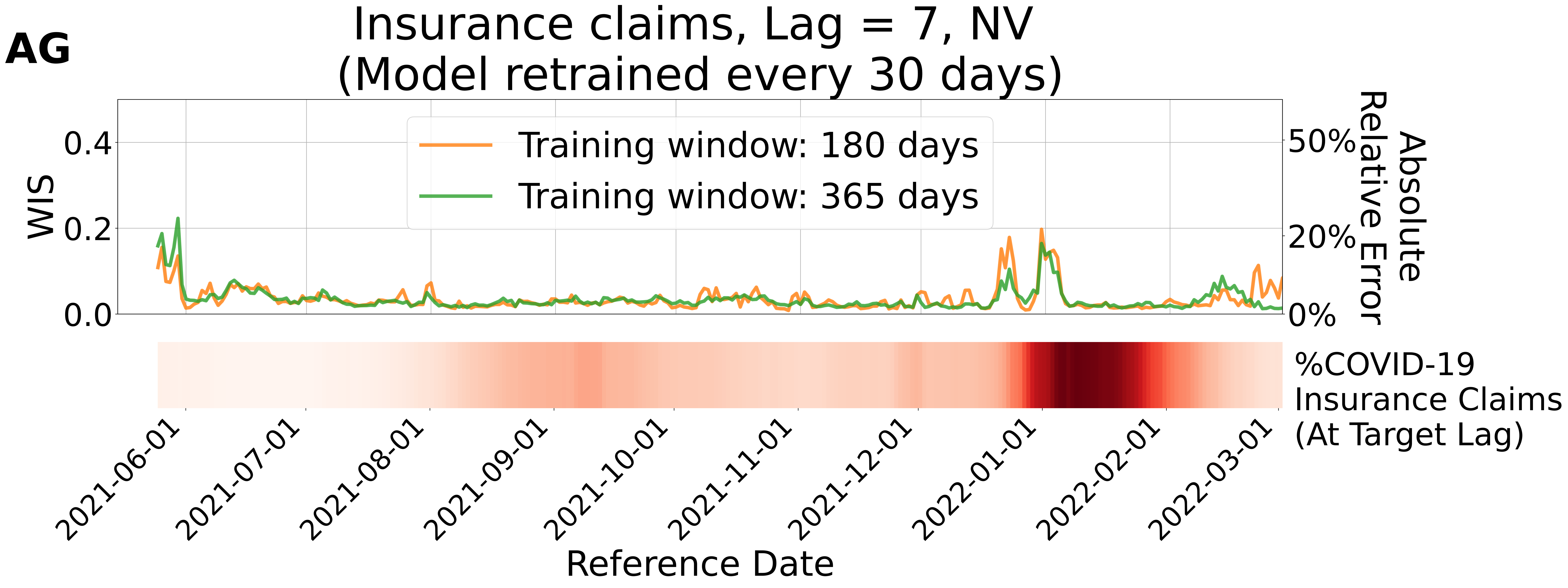
AG
Insurance claims, Lag = 7, NV
(Model retrained every 30 days)
Training window: 180 days
Training window: 365 days
WIS
0.0
0.2
0.4
Absolute Relative Error
0%
20%
50%
%COVID-19 Insurance Claims (At Target Lag)
2021-06-01
2021-07-01
2021-08-01
2021-09-01
2021-10-01
2021-11-01
2021-12-01
2022-01-01
2022-02-01
2022-03-01
Reference Date

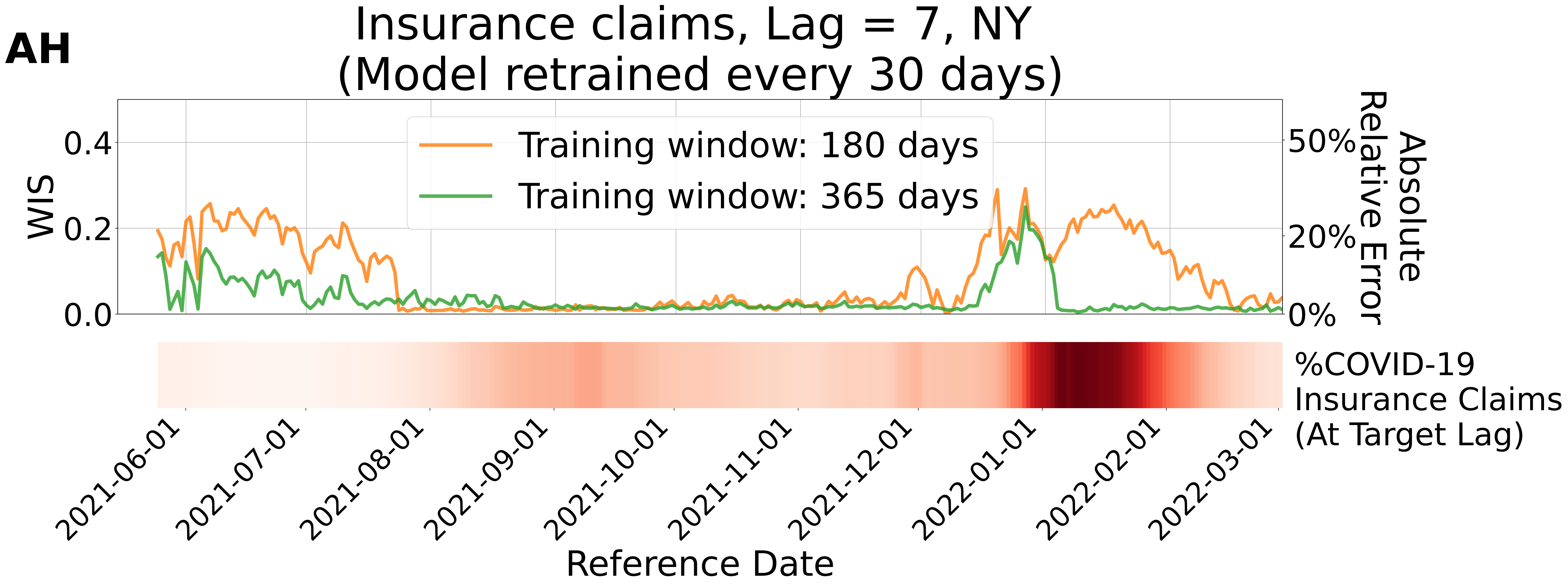
AH
Insurance claims, Lag = 7, NY
(Model retrained every 30 days)
Training window: 180 days
Training window: 365 days
WIS
0.0
0.2
0.4
Absolute Relative Error
0%
20%
50%
%COVID-19 Insurance Claims (At Target Lag)
2021-06-01
2021-07-01
2021-08-01
2021-09-01
2021-10-01
2021-11-01
2021-12-01
2022-01-01
2022-02-01
2022-03-01
Reference Date

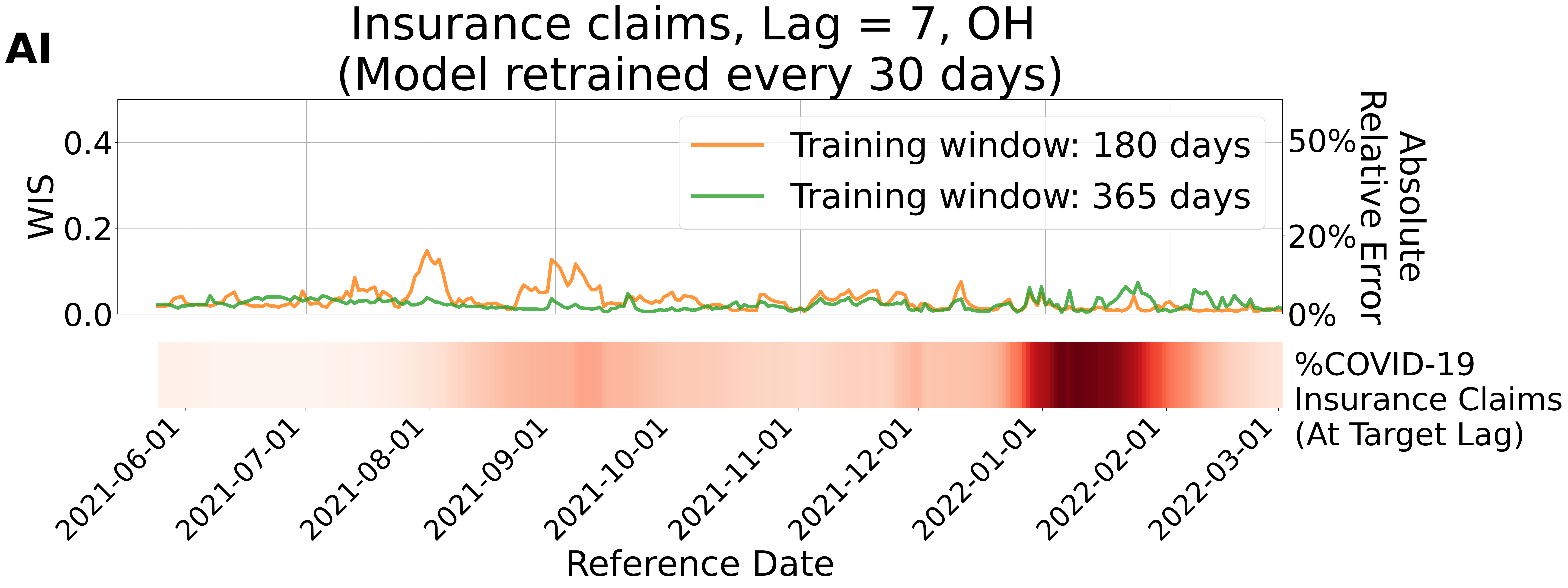

AI
Insurance claims, Lag = 7, OH
(Model retrained every 30 days)
WIS
0.0
0.2
0.4
Absolute Relative Error
0%
20%
50%
Training window: 180 days
Training window: 365 days
%COVID-19 Insurance Claims (At Target Lag)
2021-06-01
2021-07-01
2021-08-01
2021-09-01
2021-10-01
2021-11-01
2021-12-01
2022-01-01
2022-02-01
2022-03-01
Reference Date

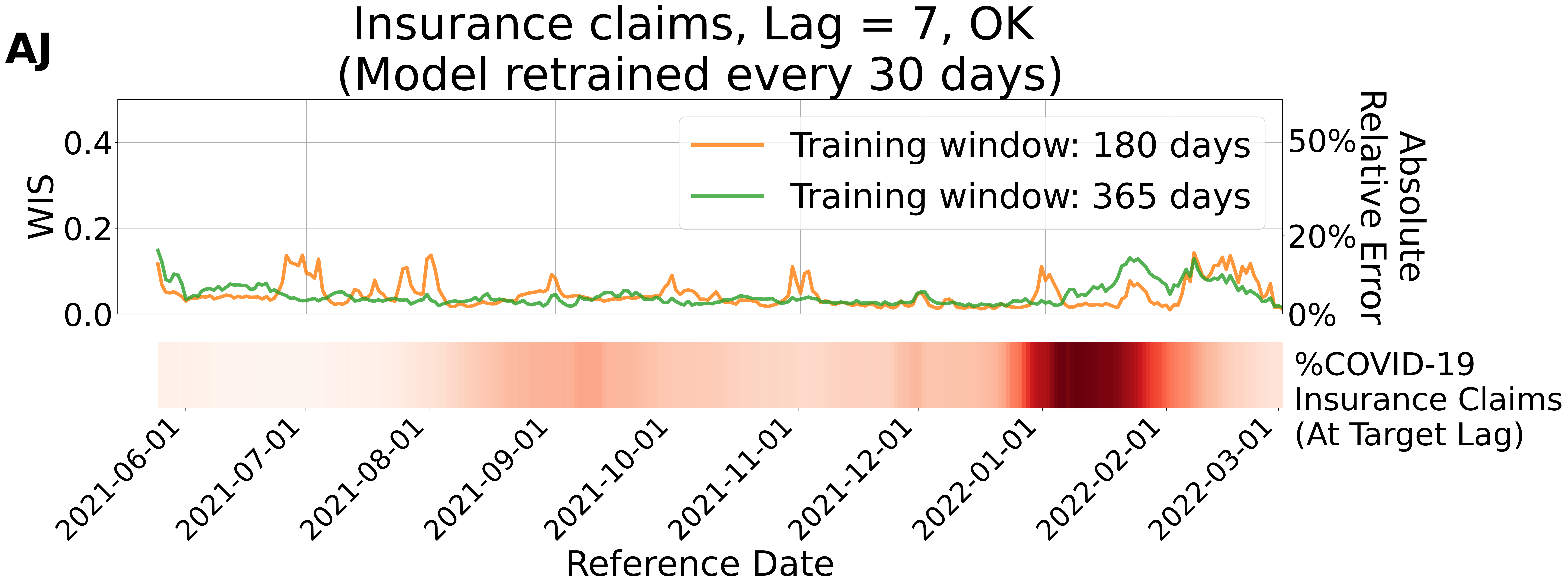

AJ
Insurance claims, Lag = 7, OK
(Model retrained every 30 days)
WIS
0.4
0.2
0.0
Absolute Relative Error
50%
20%
0%
Training window: 180 days
Training window: 365 days
%COVID-19 Insurance Claims (At Target Lag)
2021-06-01
2021-07-01
2021-08-01
2021-09-01
2021-10-01
2021-11-01
2021-12-01
2022-01-01
2022-02-01
2022-03-01
Reference Date

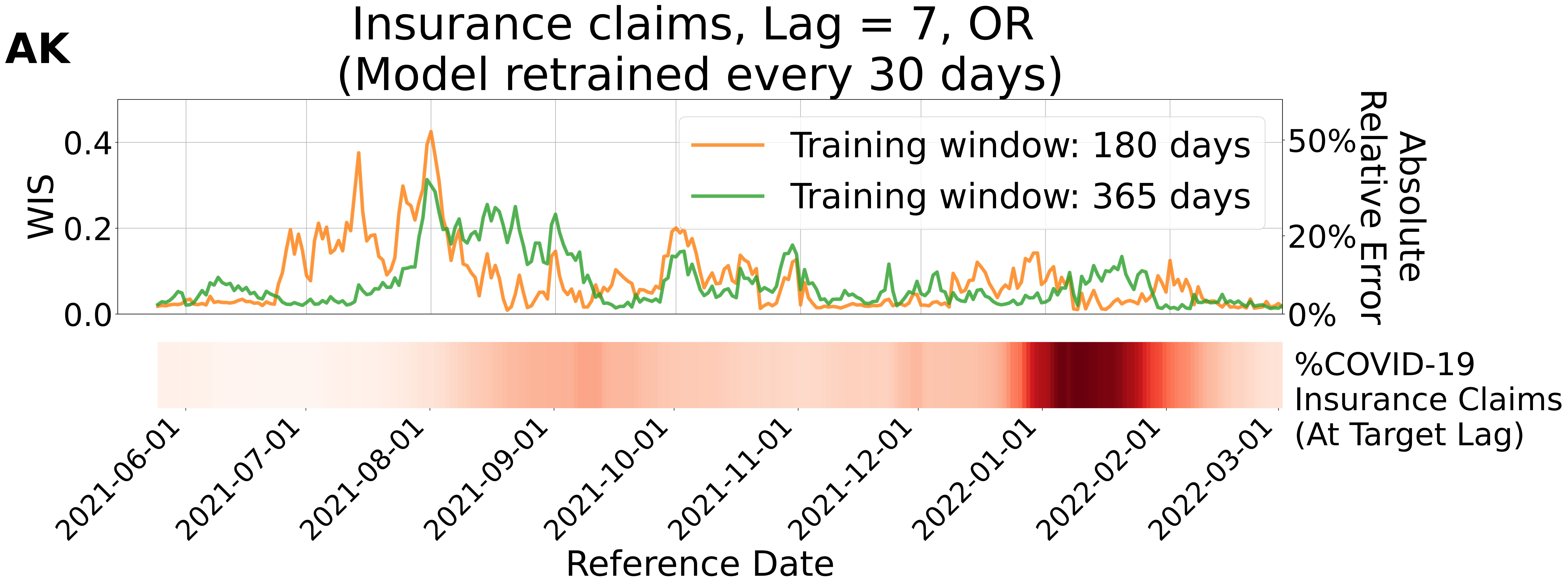

AK
Insurance claims, Lag = 7, OR
(Model retrained every 30 days)
WIS
0.0
0.2
0.4
Absolute Relative Error
0%
20%
50%
Training window: 180 days
Training window: 365 days
%COVID-19 Insurance Claims (At Target Lag)
2021-06-01
2021-07-01
2021-08-01
2021-09-01
2021-10-01
2021-11-01
2021-12-01
2022-01-01
2022-02-01
2022-03-01
Reference Date

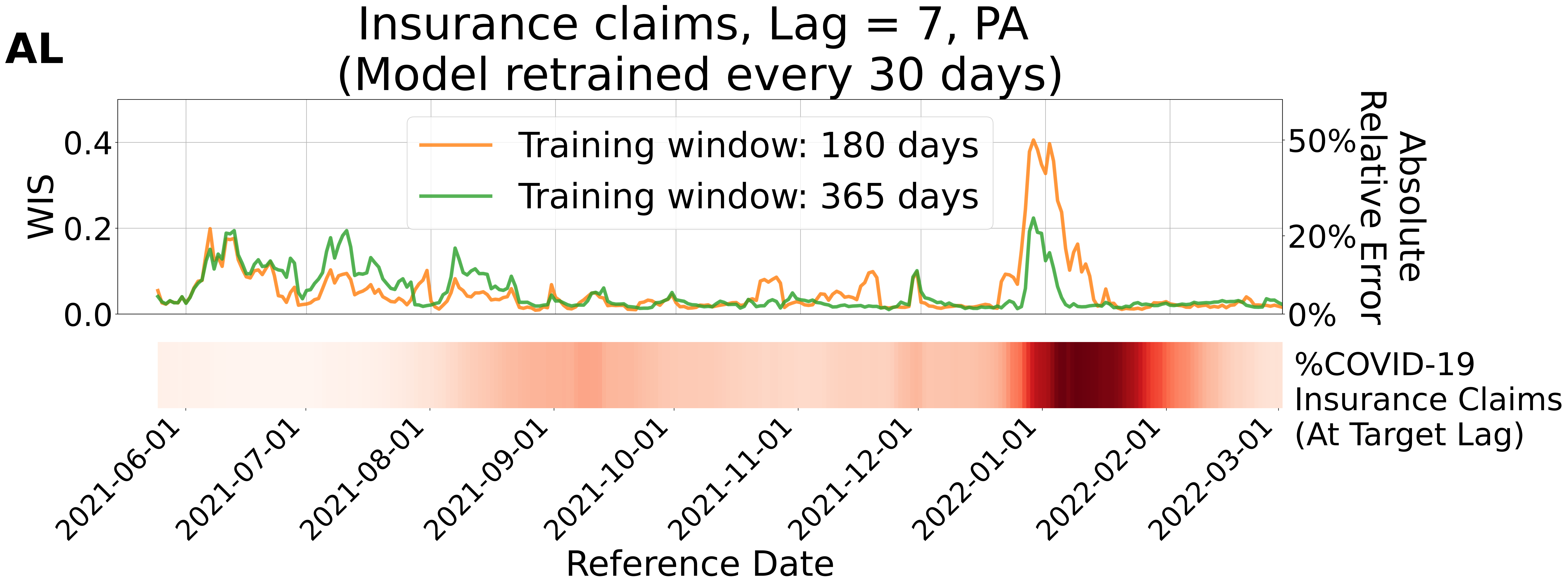

AL
Insurance claims, Lag = 7, PA
(Model retrained every 30 days)
WIS
0.0
0.2
0.4
Absolute Relative Error
0%
20%
50%
Training window: 180 days
Training window: 365 days
%COVID-19 Insurance Claims (At Target Lag)
2021-06-01
2021-07-01
2021-08-01
2021-09-01
2021-10-01
2021-11-01
2021-12-01
2022-01-01
2022-02-01
2022-03-01
Reference Date

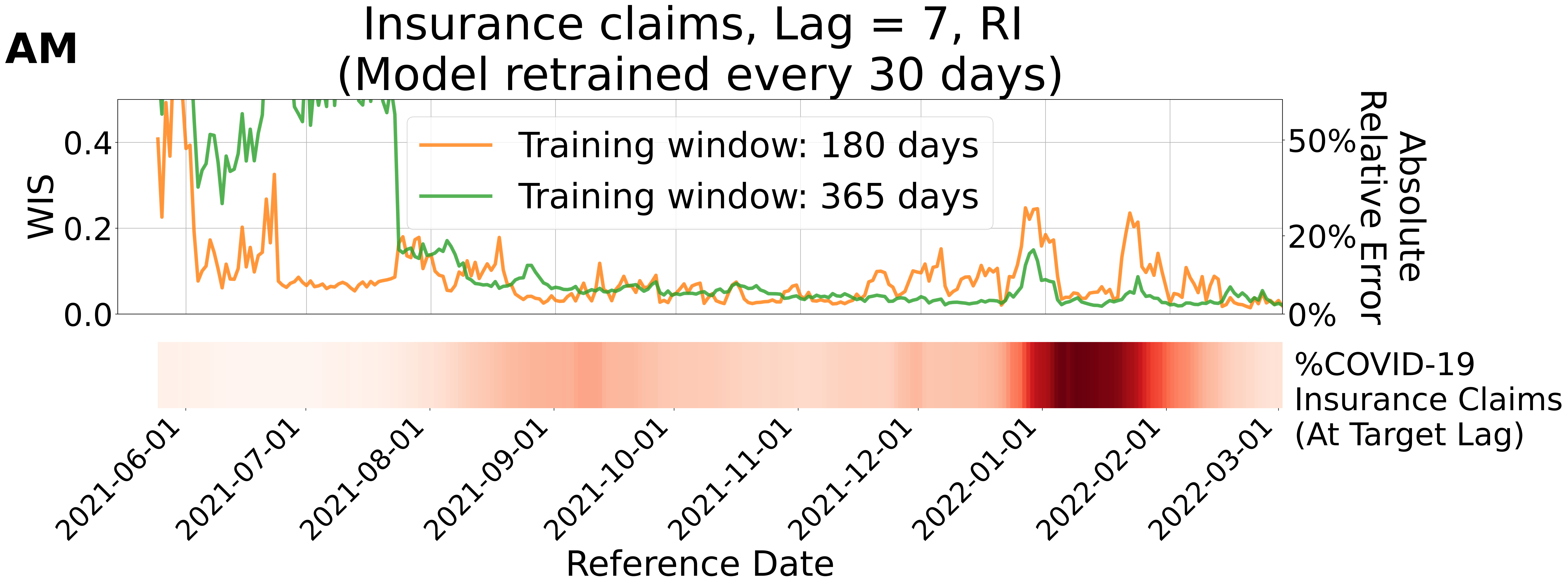
AM
Insurance claims, Lag = 7, RI
(Model retrained every 30 days)
Training window: 180 days
Training window: 365 days
WIS
0.0
0.2
0.4
Absolute Relative Error
0%
20%
50%
%COVID-19 Insurance Claims (At Target Lag)
2021-06-01
2021-07-01
2021-08-01
2021-09-01
2021-10-01
2021-11-01
2021-12-01
2022-01-01
2022-02-01
2022-03-01
Reference Date

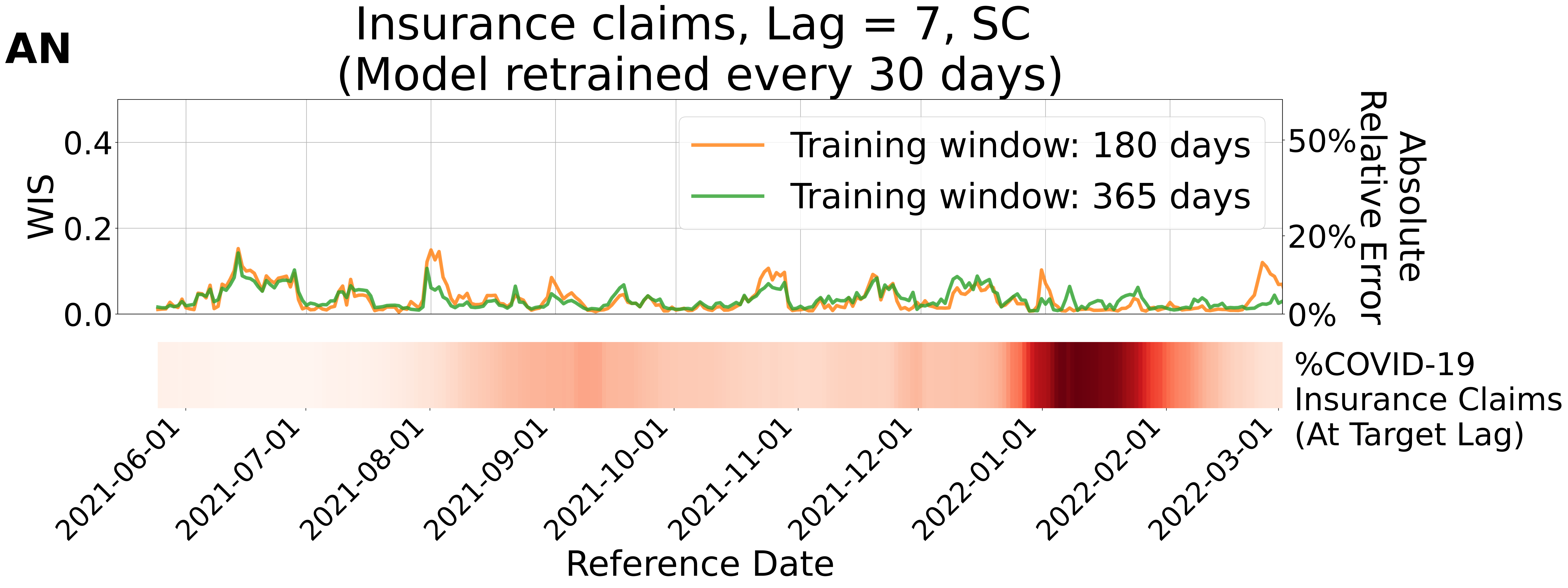
AN
Insurance claims, Lag = 7, SC
(Model retrained every 30 days)
WIS
0.4
0.2
0.0
Absolute Relative Error
50%
20%
0%
Training window: 180 days
Training window: 365 days
%COVID-19
Insurance Claims
(At Target Lag)
2021-06-01
2021-07-01
2021-08-01
2021-09-01
2021-10-01
2021-11-01
2021-12-01
2022-01-01
2022-02-01
2022-03-01
Reference Date

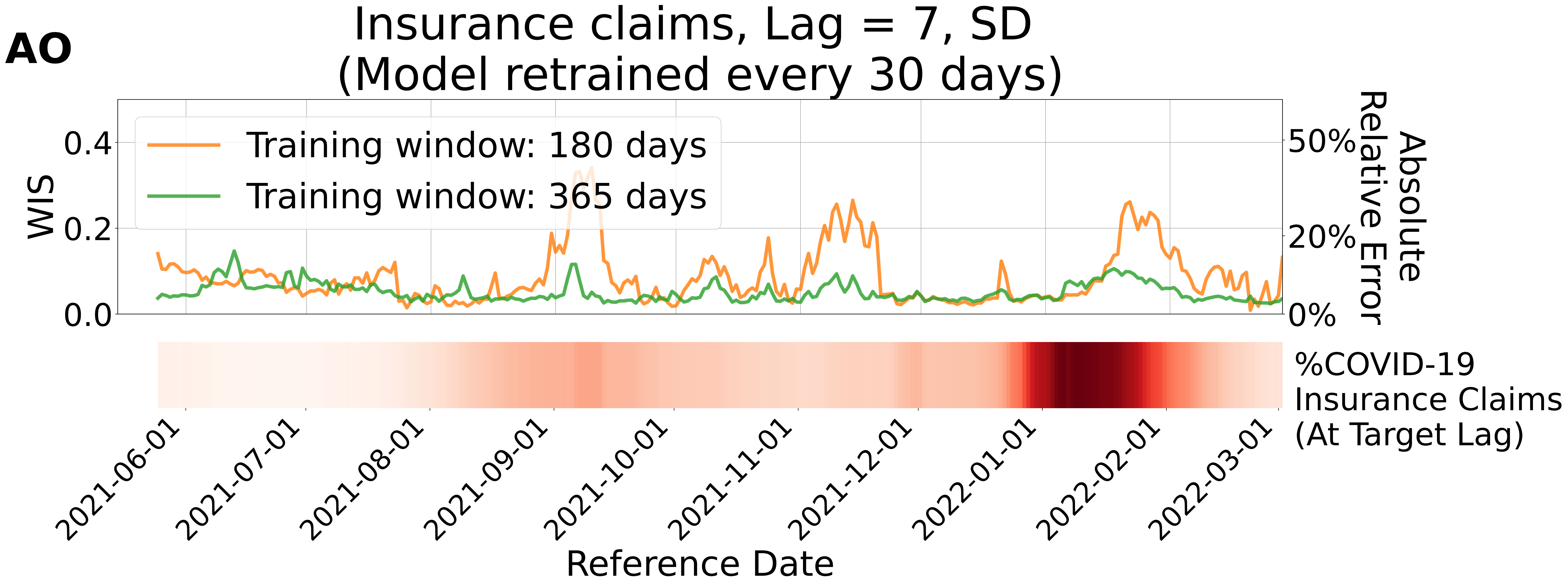

AO
Insurance claims, Lag = 7, SD
(Model retrained every 30 days)
Training window: 180 days
Training window: 365 days
WIS
0.0
0.2
0.4
Absolute Relative Error
0%
20%
50%
%COVID-19 Insurance Claims (At Target Lag)
2021-06-01
2021-07-01
2021-08-01
2021-09-01
2021-10-01
2021-11-01
2021-12-01
2022-01-01
2022-02-01
2022-03-01
Reference Date

AP

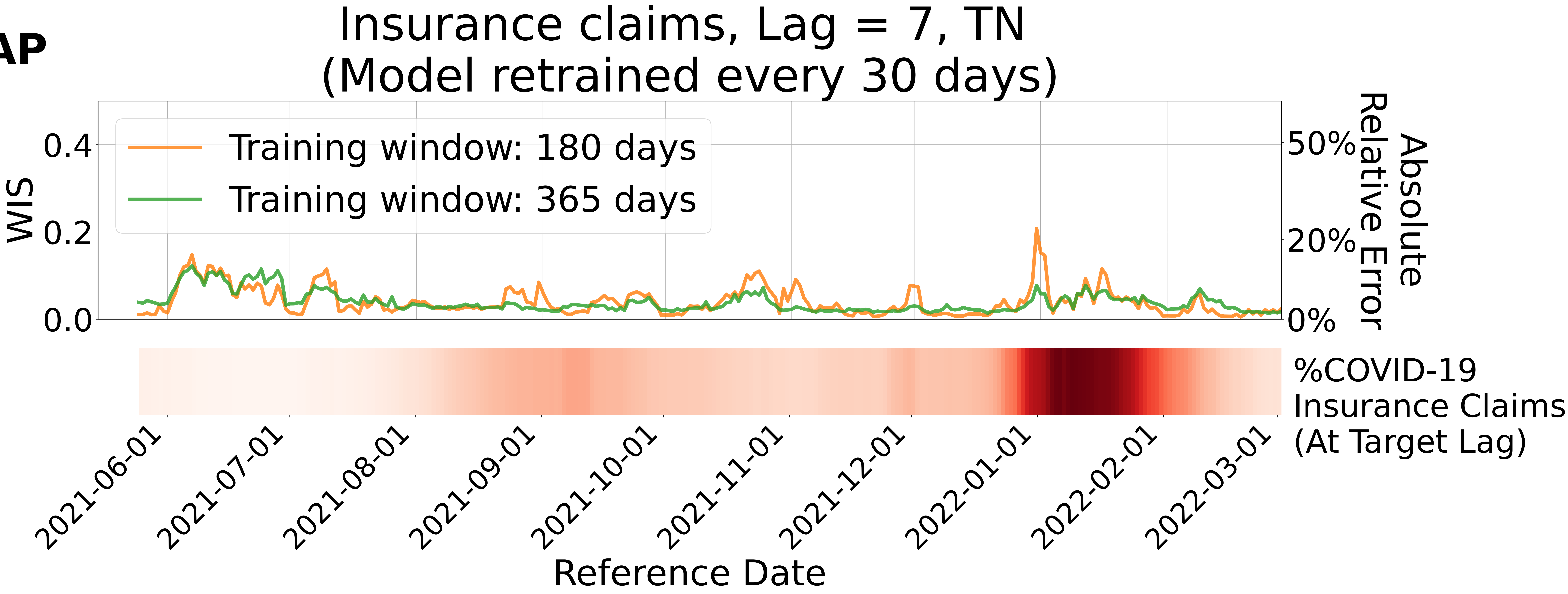

Insurance claims, Lag = 7, TN
(Model retrained every 30 days)
Training window: 180 days
Training window: 365 days
WIS
0.4
0.2
0.0
Absolute Relative Error
50%
20%
0%
%COVID-19 Insurance Claims (At Target Lag)
2021-06-01
2021-07-01
2021-08-01
2021-09-01
2021-10-01
2021-11-01
2021-12-01
2022-01-01
2022-02-01
2022-03-01
Reference Date

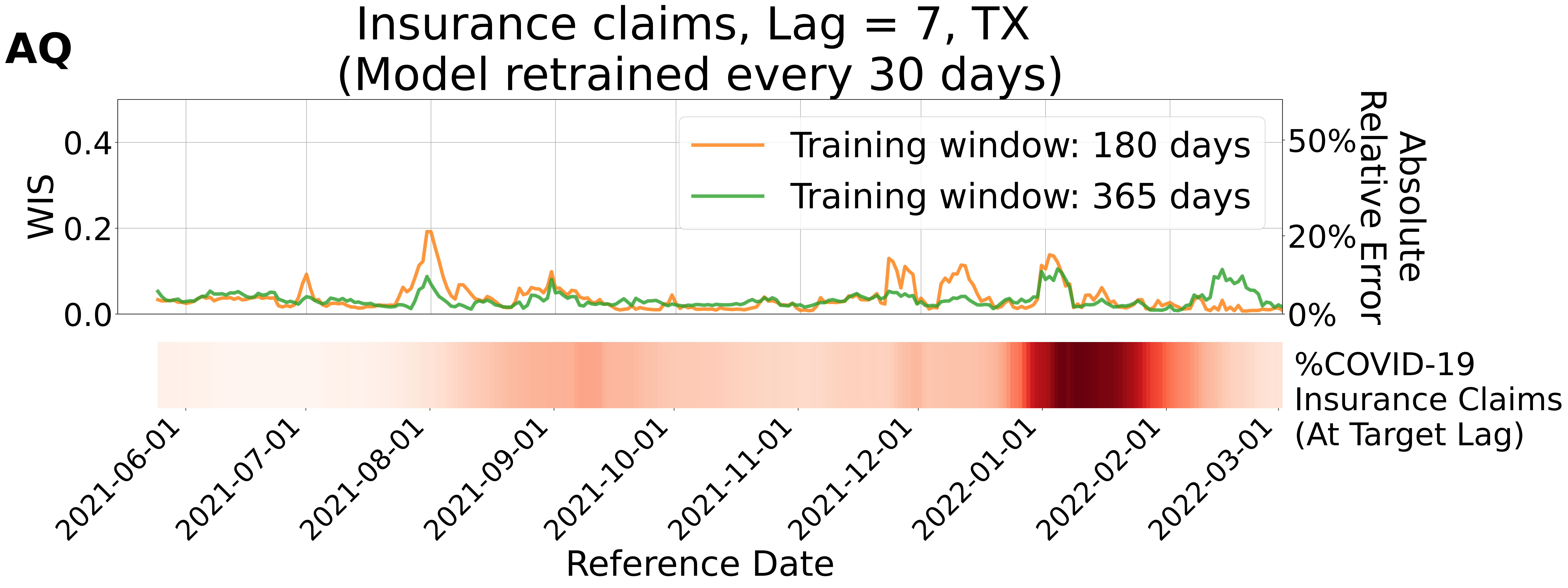
AQ
Insurance claims, Lag = 7, TX
(Model retrained every 30 days)
WIS
0.0
0.2
0.4
Absolute Relative Error
0%
20%
50%
Training window: 180 days
Training window: 365 days
%COVID-19 Insurance Claims (At Target Lag)
2021-06-01
2021-07-01
2021-08-01
2021-09-01
2021-10-01
2021-11-01
2021-12-01
2022-01-01
2022-02-01
2022-03-01
Reference Date

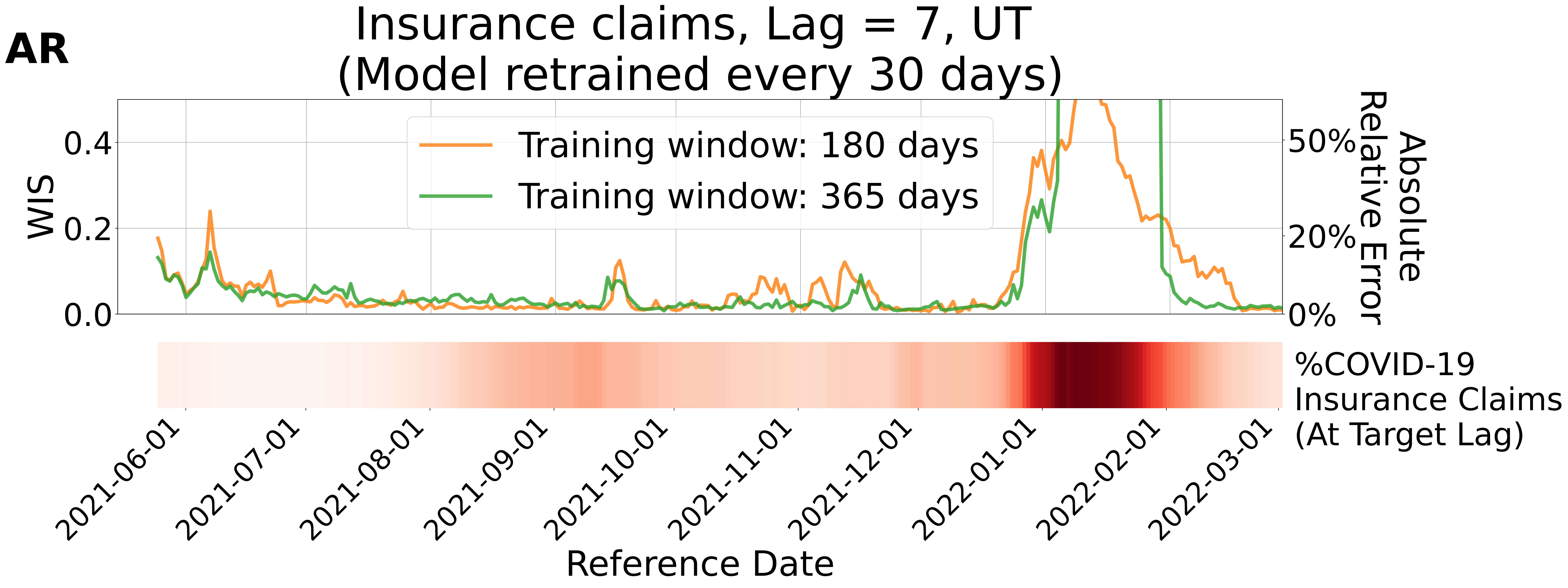
AR
Insurance claims, Lag = 7, UT
(Model retrained every 30 days)
Training window: 180 days
Training window: 365 days
WIS
0.0
0.2
0.4
Absolute Relative Error
0%
20%
50%
%COVID-19 Insurance Claims (At Target Lag)
2021-06-01
2021-07-01
2021-08-01
2021-09-01
2021-10-01
2021-11-01
2021-12-01
2022-01-01
2022-02-01
2022-03-01
Reference Date

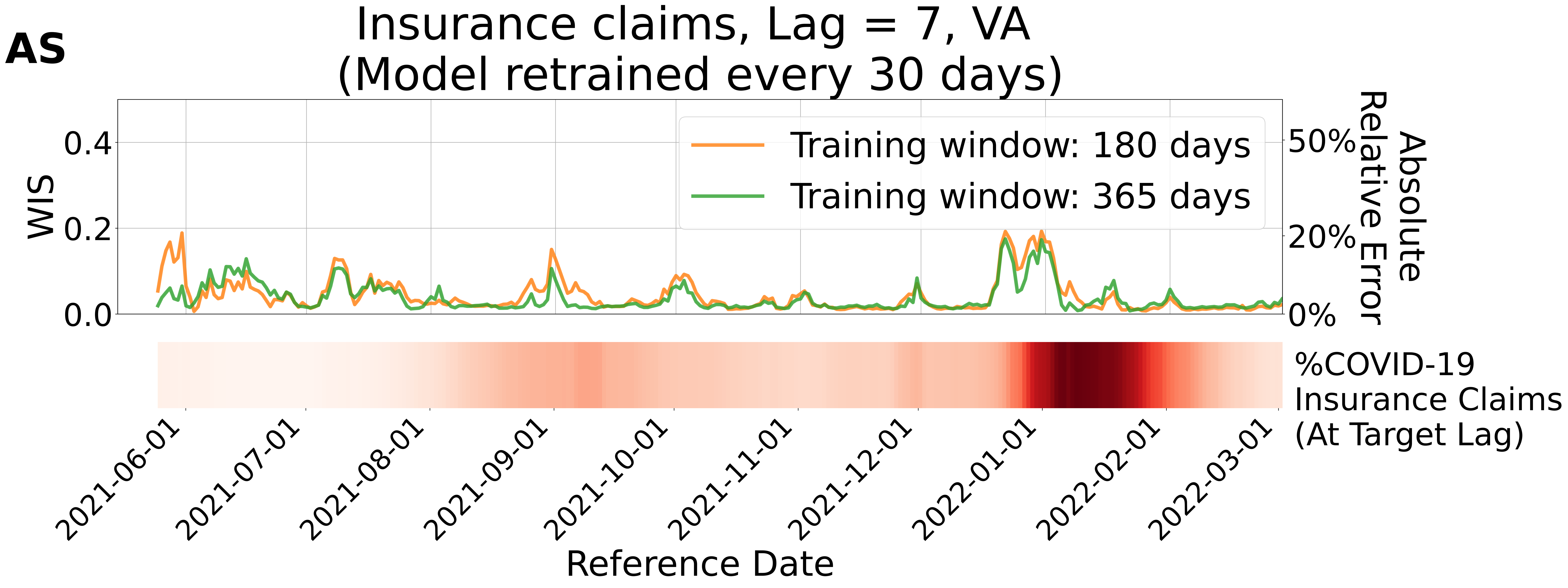

AS
Insurance claims, Lag = 7, VA
(Model retrained every 30 days)
WIS
0.0
0.2
0.4
Absolute Relative Error
0%
20%
50%
Training window: 180 days
Training window: 365 days
%COVID-19 Insurance Claims (At Target Lag)
2021-06-01
2021-07-01
2021-08-01
2021-09-01
2021-10-01
2021-11-01
2021-12-01
2022-01-01
2022-02-01
2022-03-01
Reference Date

AT

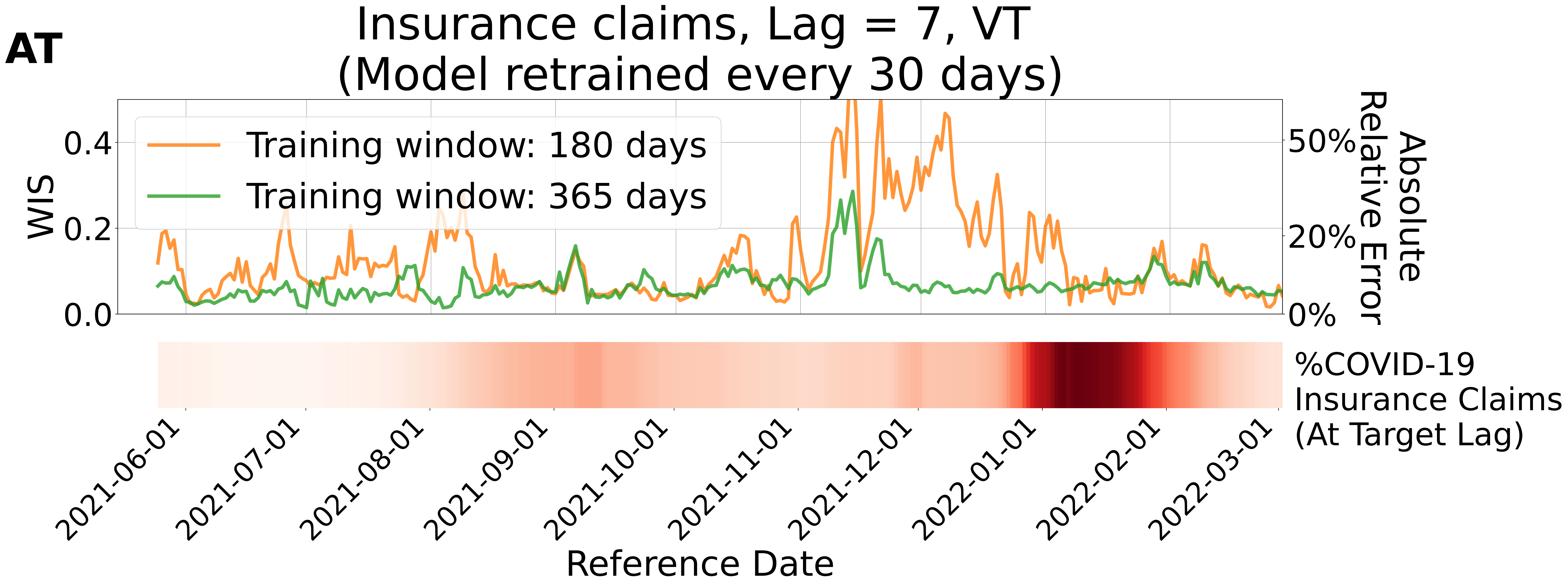

Insurance claims, Lag = 7, VT
(Model retrained every 30 days)
Training window: 180 days
Training window: 365 days
WIS
0.0
0.2
0.4
Absolute Relative Error
0%
20%
50%
%COVID-19 Insurance Claims (At Target Lag)
2021-06-01
2021-07-01
2021-08-01
2021-09-01
2021-10-01
2021-11-01
2021-12-01
2022-01-01
2022-02-01
2022-03-01
Reference Date

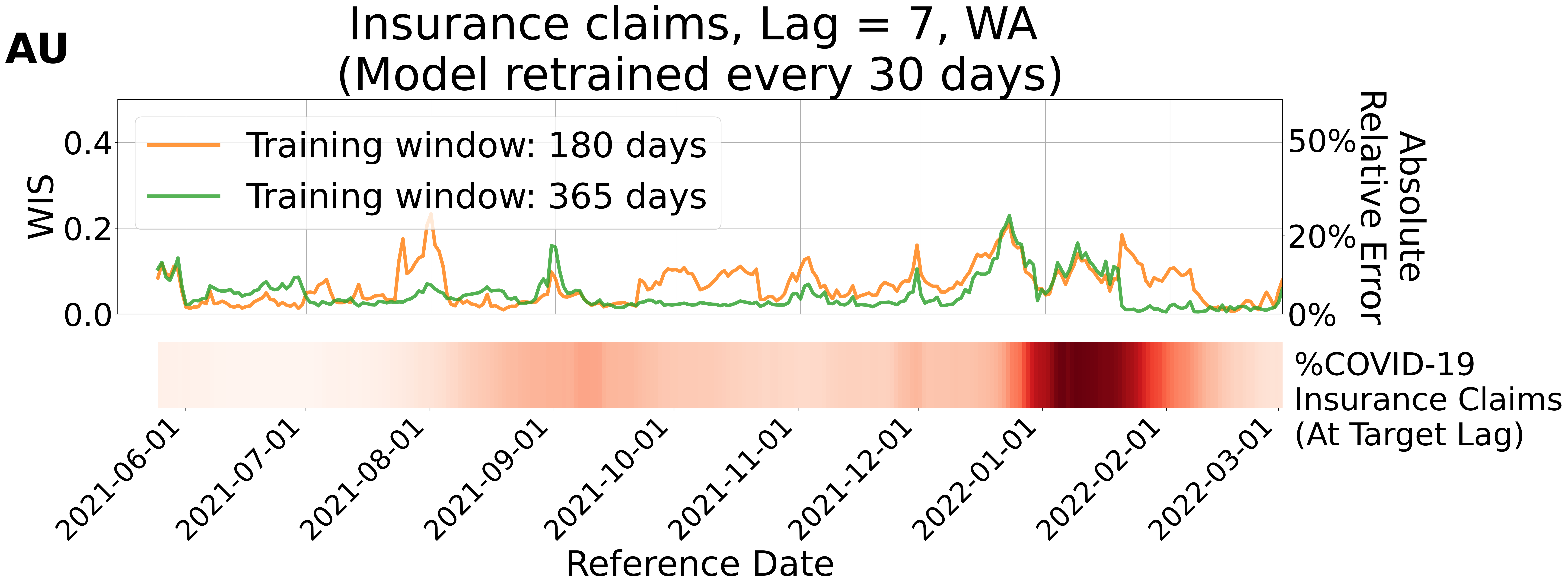

AU
Insurance claims, Lag = 7, WA
(Model retrained every 30 days)
WIS
0.0
0.2
0.4
Absolute Relative Error
0%
20%
50%
Training window: 180 days
Training window: 365 days
%COVID-19 Insurance Claims (At Target Lag)
2021-06-01
2021-07-01
2021-08-01
2021-09-01
2021-10-01
2021-11-01
2021-12-01
2022-01-01
2022-02-01
2022-03-01
Reference Date

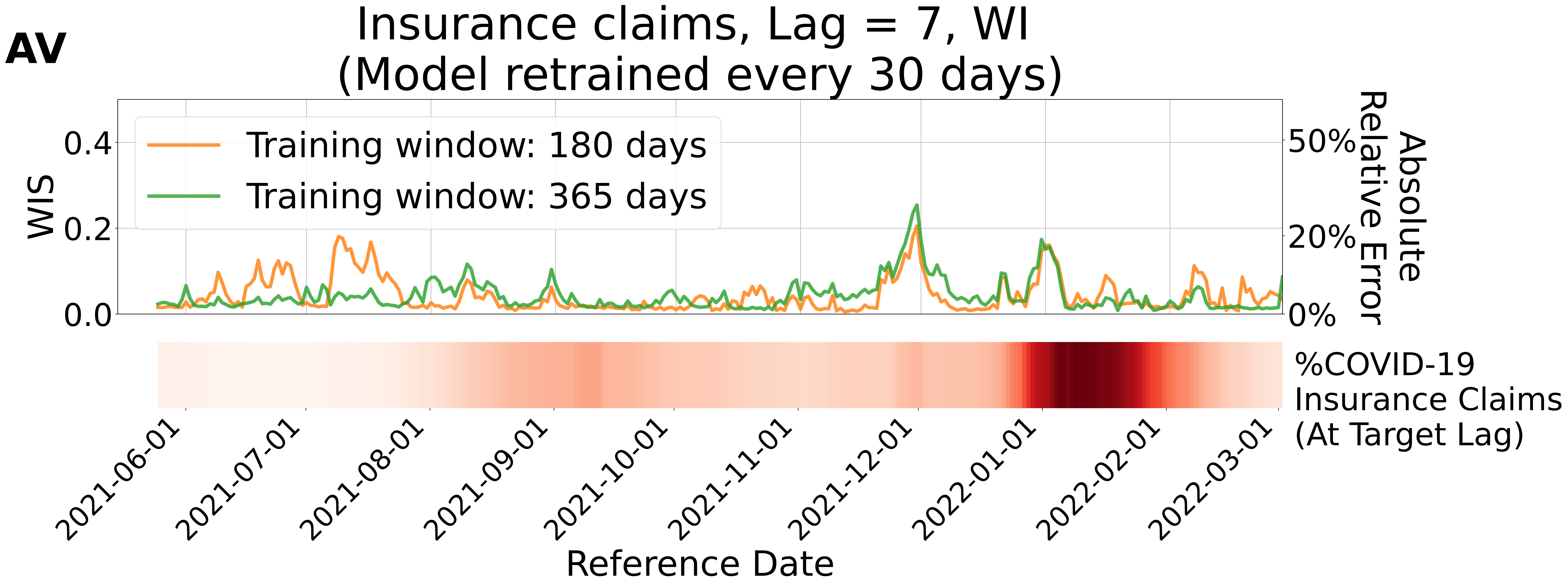
AV
Insurance claims, Lag = 7, WI
(Model retrained every 30 days)
Training window: 180 days
Training window: 365 days
WIS
0.0
0.2
0.4
Absolute Relative Error
0%
20%
50%
%COVID-19
Insurance Claims
(At Target Lag)
2021-06-01
2021-07-01
2021-08-01
2021-09-01
2021-10-01
2021-11-01
2021-12-01
2022-01-01
2022-02-01
2022-03-01
Reference Date

AW

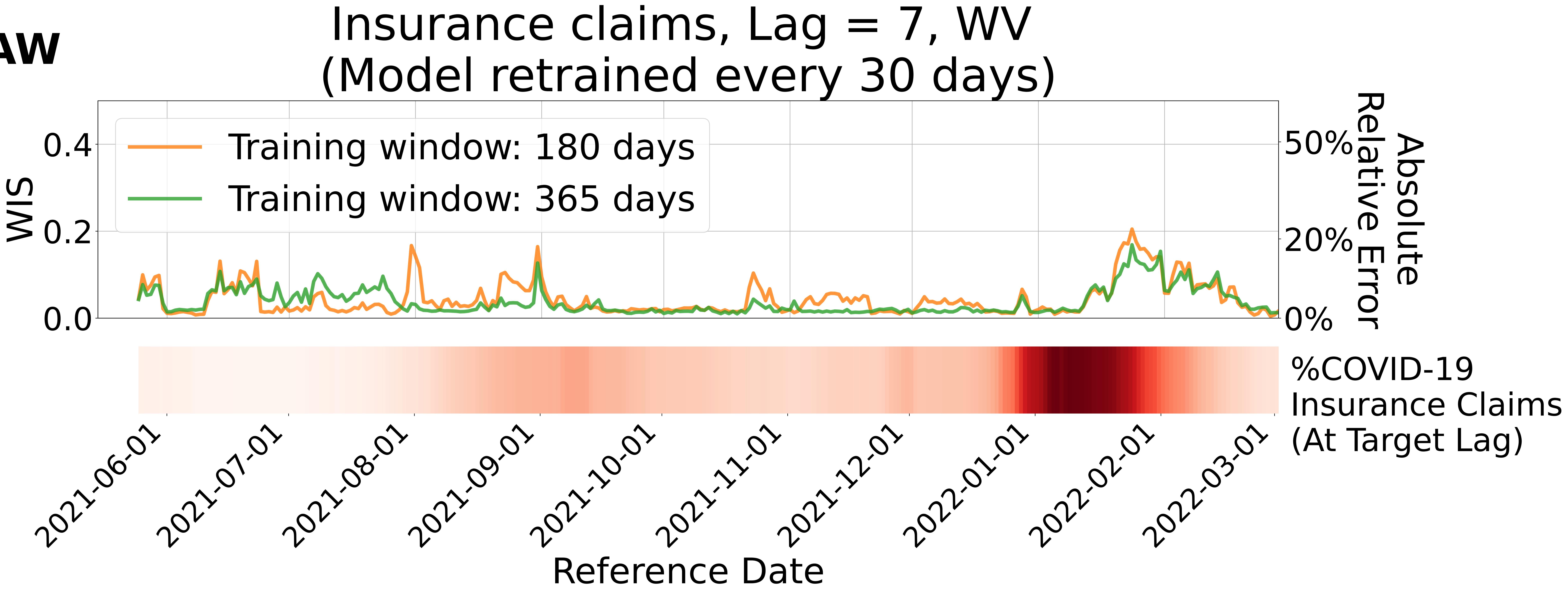

Insurance claims, Lag = 7, WV
(Model retrained every 30 days)
Training window: 180 days
Training window: 365 days
WIS
0.0
0.2
0.4
Absolute Relative Error
0%
20%
50%
%COVID-19 Insurance Claims (At Target Lag)
2021-06-01
2021-07-01
2021-08-01
2021-09-01
2021-10-01
2021-11-01
2021-12-01
2022-01-01
2022-02-01
2022-03-01
Reference Date

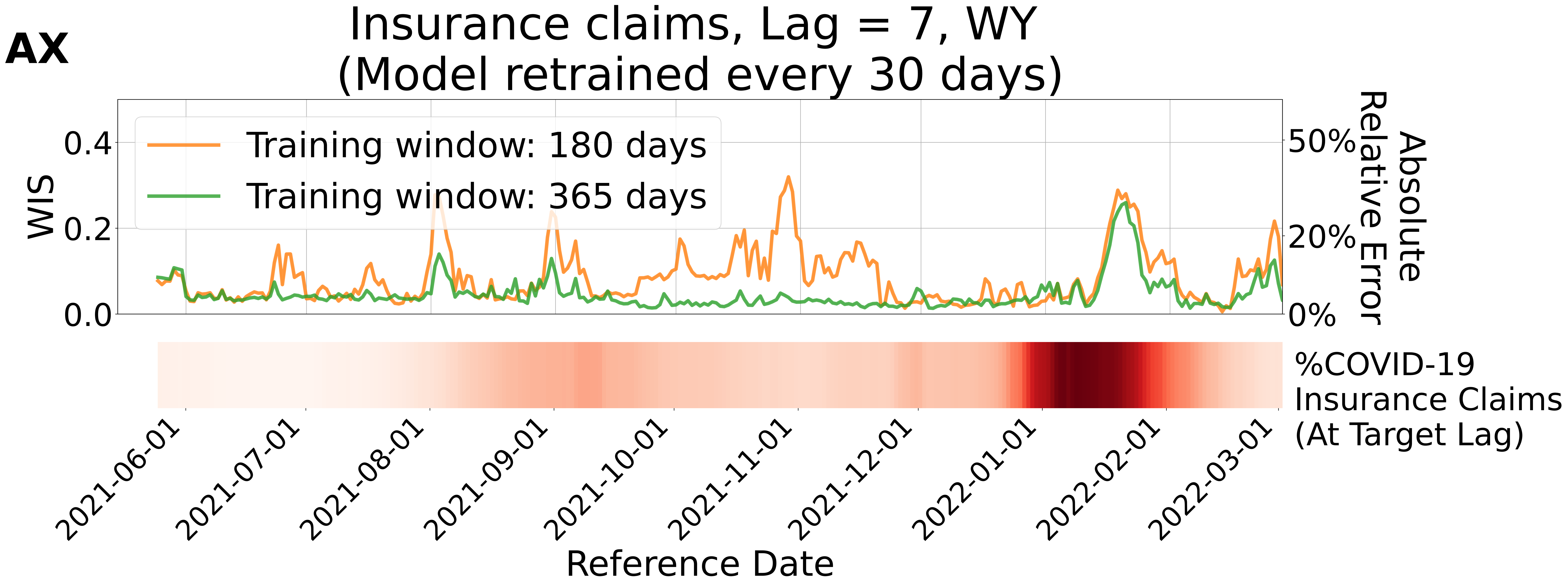
AX
Insurance claims, Lag = 7, WY
(Model retrained every 30 days)
Training window: 180 days
Training window: 365 days
WIS
0.0
0.2
0.4
0%
20%
50%
Absolute
Relative Error
%COVID-19
Insurance Claims
(At Target Lag)
2021-06-01
2021-07-01
2021-08-01
2021-09-01
2021-10-01
2021-11-01
2021-12-01
2022-01-01
2022-02-01
2022-03-01
Reference Date
