# Supplementary figures and images for: Real-time forecasting of data revisions in epidemic surveillance streams
Source: PLoS Comput Biol. 2025 Nov 20;21(11):e1013709. doi: 10.1371/journal.pcbi.1013709 (PMC12646461; doi:10.1371/journal.pcbi.1013709)

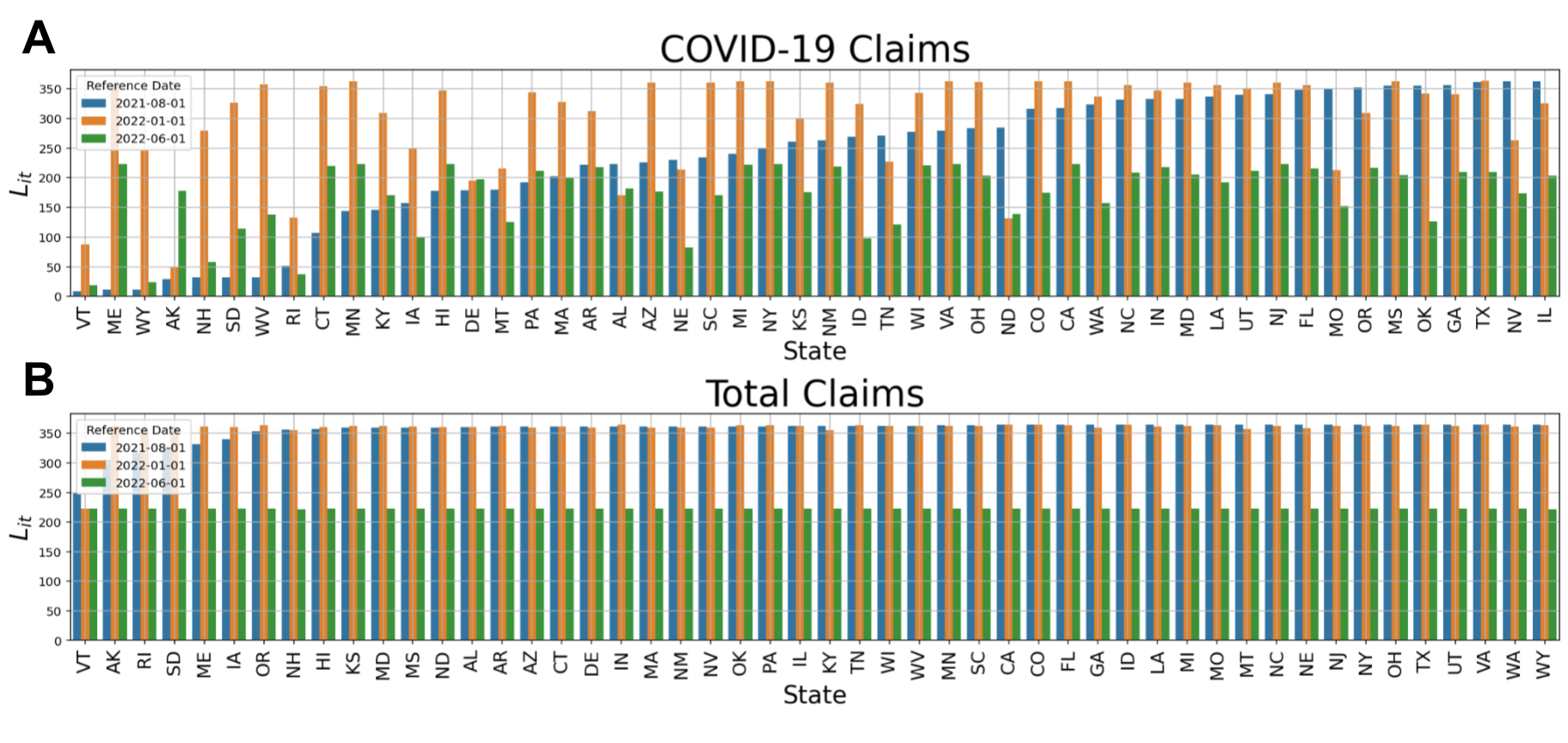

Supplement: S1 Fig — (A) Comparison of the lags required for convergence across states, shown for a sample of different reference dates based on CHNG outpatient COVID-19 insurance claims. (B) Same as in (A), but based on CHNG outpatient total insurance claims data. (TIFF) [file pcbi.1013709.s003.tiff]

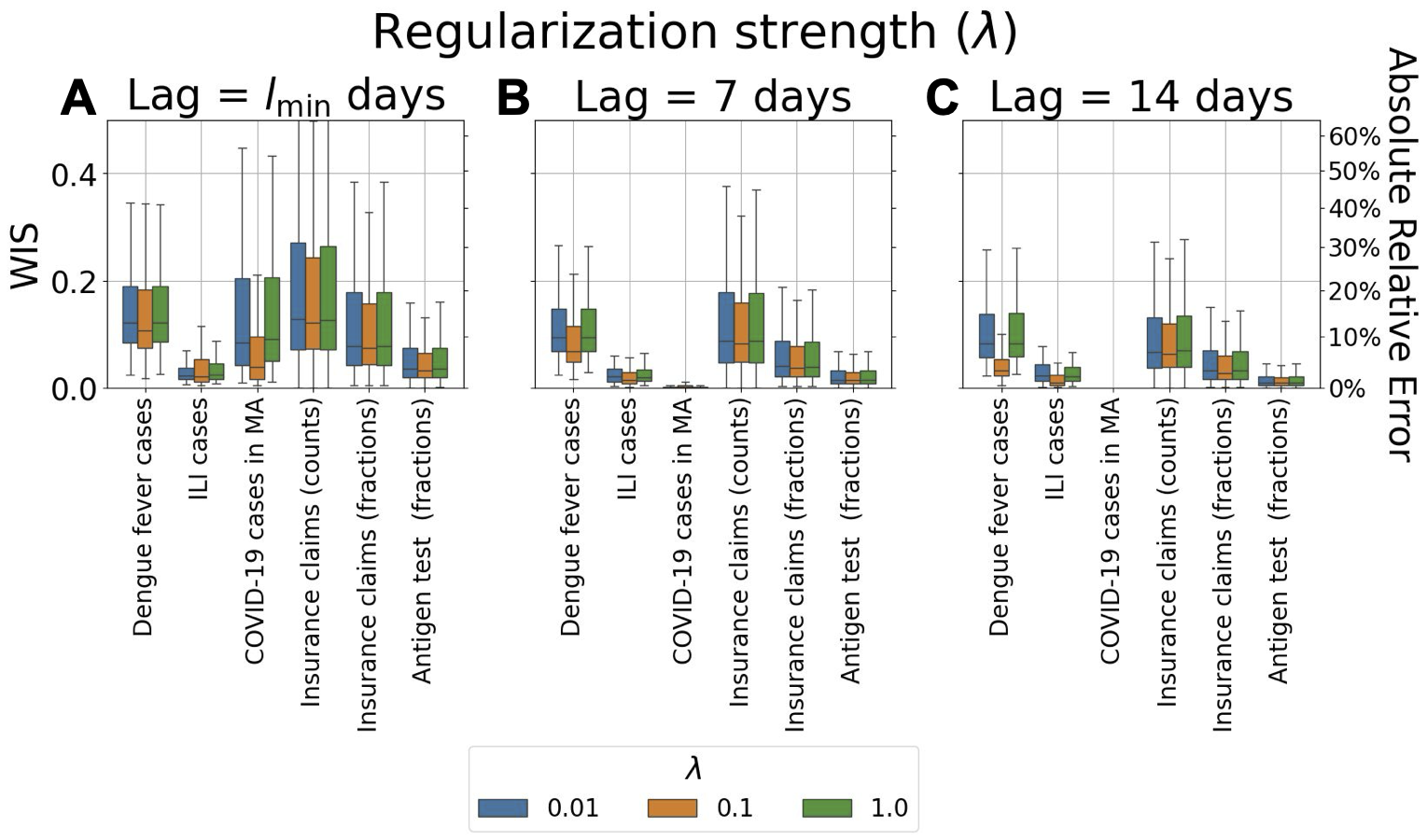

Supplement: S3 Fig — (TIFF) [file pcbi.1013709.s005.tiff]

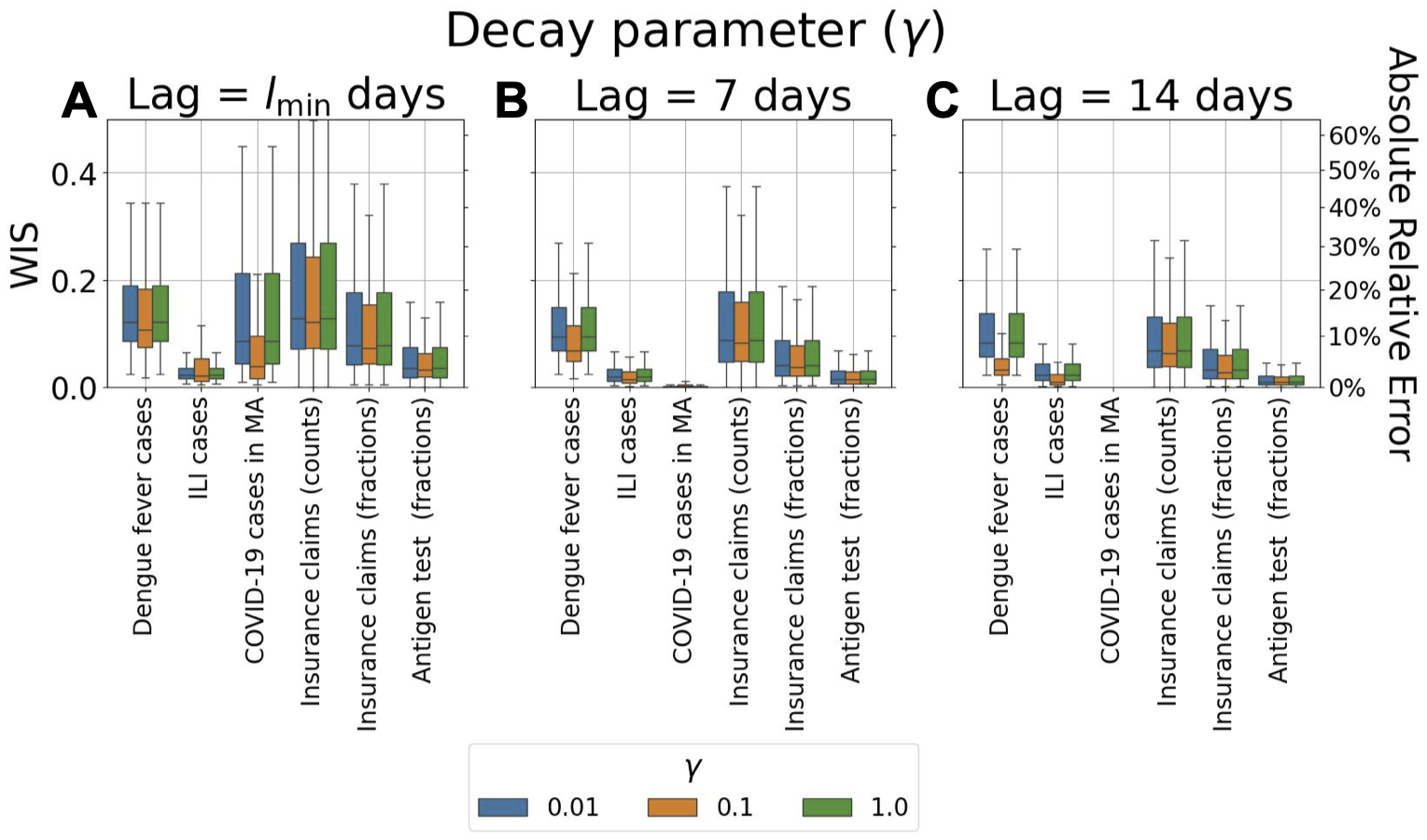

Supplement: S4 Fig — (TIFF) [file pcbi.1013709.s006.tiff]

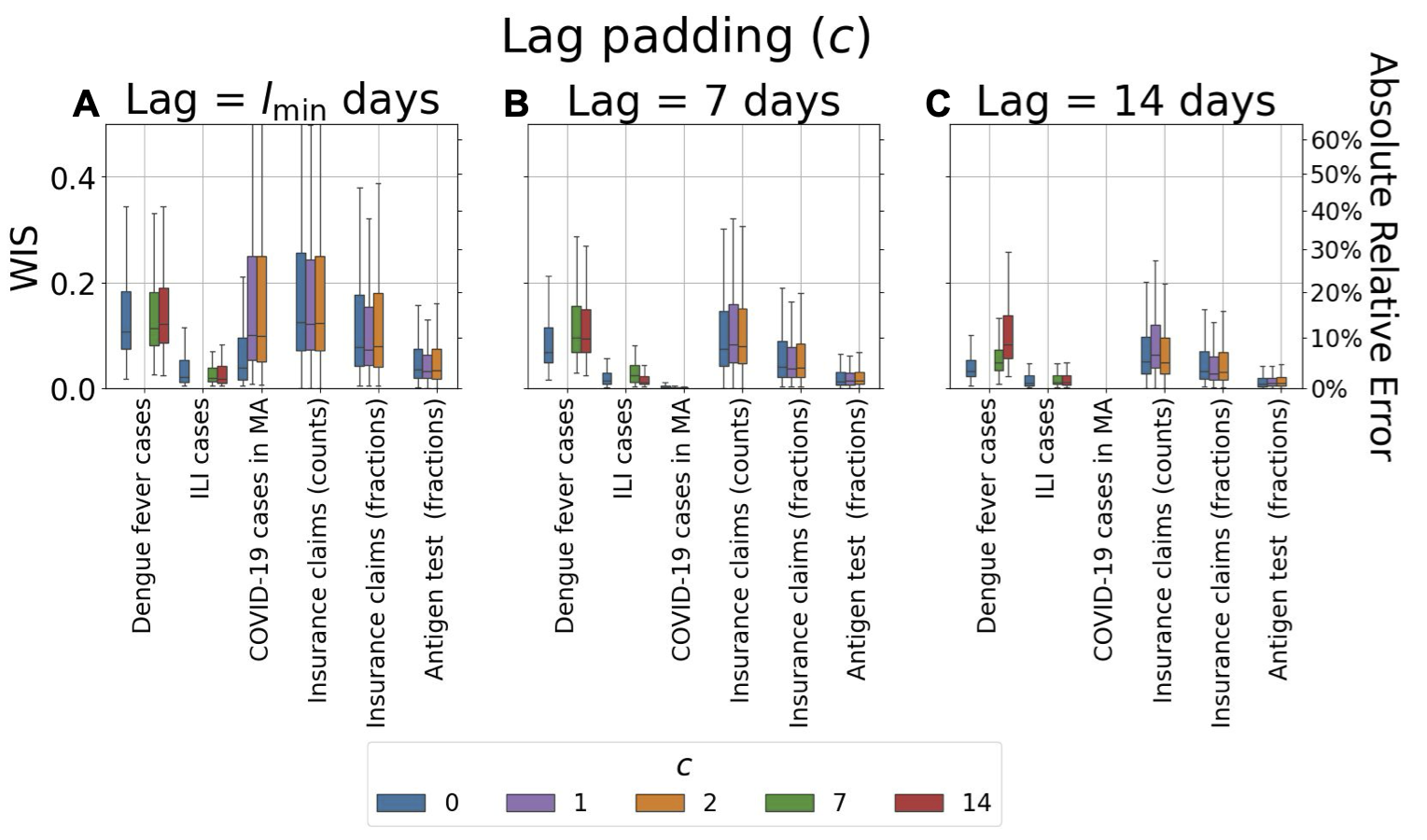

Supplement: S5 Fig — (TIFF) [file pcbi.1013709.s007.tiff]
