# Supplementary material for: Real-time forecasting of data revisions in epidemic surveillance streams
Source: PLoS Comput Biol. 2025 Nov 20;21(11):e1013709. doi: 10.1371/journal.pcbi.1013709 (PMC12646461; doi:10.1371/journal.pcbi.1013709)
Supplement: Attachment — Submitted filename: Review_D_25_00938.pdf [file pcbi.1013709.s008.pdf]

Thank you for inviting me to review “Real-time Forecasting of Data Revisions in Epidemic Surveillance Streams.” This paper is well written and allows for scientific scrutiny. It is important to emphasise that, while my work focuses on infectious disease modelling, I acknowledge that forecasting is not my area of expertise. Therefore, I will keep this review relatively brief to avoid doing more harm than good. After reading the document, I have the following **major concerns**:

1. In the introduction, it is stated: *“This limitation arises from the fact that Bayesian methods are generally built on assumptions of an underlying mechanistic process for how count data evolve over time, an assumption that does not hold for many fraction-type data, which often lack a clear generative structure.”* I understand that a problem can be approached in different ways, and this paper proposes a regression model. However, stating that *“fraction data”* lack a clear generative structure is somewhat of a stretch. In science, we have developed theories for some of the most complex phenomena imaginable, so it is reasonable to assume that causal explanations for the dynamics of fraction data can be formulated. Moreover, we have well-established tools such as fractional logistic regression, beta regression, and zero-inflated beta regression. Therefore, I suggest rephrasing this sentence and clarifying which properties of the proposed model make it particularly suitable for the problem at hand.

2. As acknowledged in the document, the Delphi RF model is not a mechanistic theory of the observed data. Rather, it is a black-box model that aims to provide accurate estimates regardless of the structural validity of its components. A crucial point for potential readers is understanding why this particular model can compete with more elaborate formulations, such as those embedded in EpiNowcast. For instance, are all the terms in the regression model necessary to achieve accurate estimates? How important is hyperparameter tuning? What are the implications of choosing the wrong value for *L* (fixed target lag)? In other words, please run sensitivity analyses that can help answer these questions. Additionally, incorporate the findings from this analysis into the conclusion/ discussion section, which currently requires further elaboration.

Minor comments:

- Could you create a schematic figure of the Delphi Revision Forecast model? It would greatly help the reader gain an overall understanding.

- Please annotate panels in Figures, and reference them accordingly in the main text. Namely, Fig 2A, Fig 2B, and so on.

- One of the most inconvenient drawbacks in the literature in the inconsistent terminology. In my view, the word "forecast" suggests predicting the future. However, in this context, the proposed model in the paper is predicting recent values, a procedure that in the infectious disease modelling literature is termed as "nowcasting". Please incorporate the footnote in the abstract into the introduction.

- Line 148. What do you mean by non-parametric?

- Overall, the methods section is devoid of references. It would be useful to justify many of the choices using the literature.

- Fix parenthesis in equations line 187.

- Lines 192-194. Please rephrase the sentence. It's difficult to read.

- Please justify the choice of imputing missing data with zeros in lines 345-347.

- Line 354. Fix typo "count-tyoe data"

- I am concerned that in the results section all locations have been pooled, but the fitting procedure was done separately. In line 481, it is said "A comprehensive set of time series forecast evaluation results for all 50 states is provided in Appendix C." Could you provide a comment on those results?

- Line 481. "Our model generally performs well". Why? Please justify.
