# Supplementary material for: Real-time forecasting of data revisions in epidemic surveillance streams
Source: PLoS Comput Biol. 2025 Nov 20;21(11):e1013709. doi: 10.1371/journal.pcbi.1013709 (PMC12646461; doi:10.1371/journal.pcbi.1013709)
Supplement: Attachment — Submitted filename: DelphiRF-rebuttal letter.docx [file pcbi.1013709.s009.docx]

**Jingjing Tang
Carnegie Mellon School of Computer Science
Computational Biology Department
jtang@andrew.cmu.edu
2025-09-01**

**Dear Editors,**

We thank you and the reviewers for the thoughtful and constructive feedback on our manuscript, *“Real-time Forecasting of Data Revisions in Epidemic Surveillance Streams.”* We appreciate the time and expertise invested in the review, and we have carefully considered all comments. Below, we respond point-by-point to each comment and describe the corresponding revisions we have made in the revised manuscript.

## **Reviewer #1**

We thank Reviewer #1 for their positive and encouraging comments regarding the motivation, clarity, and novelty of our manuscript.

**Comment:** *“The model, its features and hyperparameters are clearly explained, although a less technical summary of the model could improve readability.”*

**Response:** To improve readability, we have added Figure 1, which provides an overview of the Delphi-RF framework. The figure illustrates the preparatory steps, the underlying data structure, and the overall workflow, offering readers an accessible summary to complement the detailed technical description in the Methods section.

**Comment:** *“Fig 7 is titled ‘Boxenplot’, which to my (vague) knowledge refers to the Seaborn implementation of the ‘letter value plot’. However, they look and are described in the Figure caption like regular boxplots?”*

**Response:** Thank you for catching this. You are correct — these plots are standard boxplots, not Seaborn boxenplots. We have corrected the figure title and the caption to refer to them properly as boxplots.

## **Reviewer #2**

We are grateful for the detailed feedback, which will substantially improve the clarity and rigor of our manuscript. Below we address each point in turn.

**Major Comment 1:**
*“In the introduction, it is stated: ‘fraction-type data … lack a clear generative structure.’ … I suggest rephrasing this sentence and clarifying which properties of the proposed model make it particularly suitable for the problem at hand.”*

**Response:** We have rephrased the sentence (now in lines 66-72) and revised the paragraph below (now in lines 90-93) to clarify that, while mechanistic models for fraction-type data can certainly be formulated, the practical challenge is that such revision data often arise from administrative or behavioral processes that are difficult to capture mechanistically. We now emphasize that our regression-based model provides a flexible, distribution-free approach that can adapt to such non-standard data dynamics.

**Major Comment 2.1:**
*“A crucial point for potential readers is understanding why this particular model can compete with more elaborate formulations, such as those embedded in EpiNowcast.*

**Response:** Thanks for pointing this out. We added the following to Section 5: *“Delphi-RF competes with more elaborate nowcasting frameworks (e.g., EpiNowcast) because it uses quantile regression forests to learn nonlinear effects from a set of covariates that encode reporting behavior (day-of-week and week-of-month by issue or reference date), lagged values, recent revision magnitudes, and short-term disease activity levels. This allows the model to capture the information that parametric delay models exploit while remaining relatively robust to misspecification and distributional shifts.”*

**Major Comment 2.2:**

*For instance, are all the terms in the regression model necessary to achieve accurate estimates?”*

**Response:** We have added a new Section 4.9 presenting an ablation study, which demonstrates that each feature group contributes to forecasting performance.

**Major Comment 2.3:**
*“How important is hyperparameter tuning?”*

**Response:** We have added a new Section 4.10 on hyperparameter sensitivity analysis. In summary, hyperparameter effects in Delphi-RF are signal- and lag-dependent, underscoring the importance of aligning hyperparameter choices with the revision dynamics of each dataset. Delphi-RF is generally robust to moderate changes in the regularization strength ($\lambda$), decay parameter ($\gamma$), and training-window length ($W$), with performance remaining stable across most signals and lags. Positive lag padding ($c>0$) is beneficial when revision patterns across neighboring lags are similar, as borrowing strength from adjacent lags improves forecast stability; however, when revision patterns differ substantially across lags, padding introduces noise and degrades performance.

**Major Comment 2.4:**
*“What are the implications of choosing the wrong value for L (fixed target lag)?”*

**Response:** Thanks for pointing out this misleading part. The **target lag is not a hyperparameter**; rather, it is a design choice for defining the forecasting problem itself, as explained in the Problem Setup part in Section 2.

We set a fixed target lag because in real time we cannot know whether reports are finalized. Using the most recent reports in lieu of finalized values would introduce noise into model training, since reports for different reference dates would be at different stages of revision.

In principle, one can choose any sufficiently large target lag, and the forecasting results will not change much because the responses at those lags are already quite stable. However, setting the target lag too large means waiting unnecessarily long for the response and losing the ability to leverage informative revision patterns in newly added reports. Conversely, if the target lag is too small, the responses remain highly variable and far from finalized, leading to unstable forecasts and limiting their usefulness to end users. In practice, one should choose the smallest target lag that still approximates the asymptotic finalized values reasonably well.

**Minor Comments**

*“Could you create a schematic figure of the Delphi Revision Forecast model? It would greatly help the reader gain an overall understanding.”*
**Response:** Yes, we have added a new schematic figure(now Figure 1) illustrating the structure and workflow of the Delphi Revision Forecast framework.

*“Please annotate panels in Figures, and reference them accordingly in the main text. Namely, Fig 2A, Fig 2B, and so on.”*
**Response:** We have updated all multi-panel figures to include panel labels (A, B, C, etc.) and revised the main text to reference these labels consistently.

*“One of the most inconvenient drawbacks in the literature is the inconsistent terminology… Please incorporate the footnote in the abstract into the introduction.”*
**Response:** Done. We have added a clarifying statement in the introduction to explain our usage of “forecast” (in lines 86-90).

*“Line 148. What do you mean by non-parametric?”*
**Response:** We mean that the Delphi-RF does not assume a specific parametric form and employs quantile regression, which provides a flexible framework for modeling the conditional distribution of revision magnitudes. This allows the model to capture heterogeneous revision behavior across time, locations, and data sources without imposing restrictive assumptions . This clarification has been added (to line 161-164 now).

*“Overall, the methods section is devoid of references. It would be useful to justify many of the choices using the literature.”*
**Response:** We have added appropriate references in the Methods section to justify our methodological choices.

*“Fix parenthesis in equations line 187.”*
**Response:** Corrected.

*“Lines 192-194. Please rephrase the sentence. It’s difficult to read.”*
**Response:** We have rewritten this sentence for clarity. (Now in lines 209-211)

“Line 354. Fix typo “count-tyoe data”
**Response:** Fixed.

*“Please justify the choice of imputing missing data with zeros.”*
**Response:** Thank you for pointing this out. Our phrase *“imputing missing data with zeros”* was misleading and does not accurately describe our approach. Since we are modeling the reporting process itself, revisions or their absence are by definition observable, and hence require no imputation. To avoid confusion, we have removed the “Missing data imputation” paragraph in Section 4.2 and revised the relevant description and definition in the “Revision Dynamics” paragraph of Section 2.

*“In line 481, it is said “A comprehensive set of time series forecast evaluation results for all 50 states is provided in Appendix C. ” Could you provide a comment on those results?”*
**Response:** We have revised the manuscript to include more descriptive commentary on these results (now in lines 520-525). Specifically, we now note that *“These results confirm the overall patterns highlighted in the main text—namely, stable performance during most of the study period with degradation during periods of extreme epidemiological change, such as the Omicron wave. Some heterogeneity in absolute WIS values is observed across states, reflecting differences in population size and reporting practices.”*

*“Line 481. “Our model generally performs well” Why? Please justify.”*
**Response:** We now provide a more detailed justification referencing quantitative metrics (e.g., mean absolute error, WIS, now in lines 502-506) to support this claim.

**Again, we thank the reviewers for their valuable feedback and believe that these revisions significantly improve the clarity, rigor, and utility of the manuscript.**

Sincerely,
Jingjing Tang
On behalf of all co-authors
